# Acute stress is detrimental to heart regeneration in zebrafish

**DOI:** 10.1098/rsob.160012

**Published:** 2016-03-30

**Authors:** Pauline Sallin, Anna Jaźwińska

**Affiliations:** Department of Biology, University of Fribourg, Chemin du Musée 10, 1700 Fribourg, Switzerland

**Keywords:** non-mammalian model, myocardium, infarction, crowding, cortisol, anxiolytic

## Abstract

Psychological stress is one of the factors associated with human cardiovascular disease. Here, we demonstrate that acute perceived stress impairs the natural capacity of heart regeneration in zebrafish. Beside physical and chemical disturbances, intermittent crowding triggered an increase in cortisol secretion and blocked the replacement of fibrotic tissue with new myocardium. Pharmacological simulation of stress by pulse treatment with dexamethasone/adrenaline reproduced the regeneration failure, while inhibition of the stress response with anxiolytic drugs partially rescued the regenerative process. Impaired heart regeneration in stressed animals was associated with a reduced cardiomyocyte proliferation and with the downregulation of several genes, including *igfbp1b*, a modulator of IGF signalling. Notably, daily stress induced a decrease in Igf1r phosphorylation. As cardiomyocyte proliferation was decreased in response to IGF-1 receptor inhibition, we propose that the stress-induced cardiac regenerative failure is partially caused by the attenuation of IGF signalling. These findings indicate that the natural regenerative ability of the zebrafish heart is vulnerable to the systemic paracrine stress response.

## 1. Introduction

In most living organisms, the experience of multiple environmental and physiological challenges has driven the development of a vital stress response system. When facing a life-threatening situation, animals instinctively enter in a fight-or-flight mode to enhance the chances of their survival. In vertebrates, this innate reaction relies on the activation of the central nervous system orchestrating rapid and powerful systems to focus the energy resources on cognitive and muscular performance. Among these networks, the hypothalamus–pituitary–adrenal (HPA) axis and the sympathetic nervous system play a crucial role by, respectively, triggering the release of corticosteroids and adrenaline/noradrenaline, which are the primary stress hormones [1,2]. The neuro-endocrine stress response system is evolutionarily conserved between human and zebrafish [3–5]. The studies in fish demonstrated that cortisol and nor-/adrenaline promote multiple physiological changes, including an enhanced oxygen uptake from the gills and an increase in glucose uptake, in order to optimize task execution during environmental perturbations [3–5].

While the stress response is beneficial in the short term, a prolonged and frequent state of danger management becomes prejudicial for the organism. In certain conditions, the individual capacity to evaluate and cope with a stressful situation can be biased and the stress response becomes maladjusted. In such cases, the organism is maintained in a state of alertness with sustained activation of the sympathetic nervous system and elevation of glucocorticoid secretion. The resulting high level of stress hormones in the body disfavours different non-vital processes, such as digestion, growth, reproduction, immune defence or wound healing in fish and mammals [6–9]. Up to now, the molecular and cellular link between stress exposure and organ restoration has not been addressed.

The zebrafish provides a valuable mode of heart regeneration [10,11]. Lineage tracing experiments have demonstrated that the regenerated myocardium derives from proliferating pre-existing cardiomyocytes (CMs) [12,13]. Cardiac cells at the injury border reactivate developmental programmes, efficiently undergo mitosis and repopulate the injury zone, which only transiently heals by fibrotic tissue [12–14]. In addition to the local response at the site of injury, CM proliferation becomes globally increased within the entire myocardium without undergoing obvious dedifferentiation [11,14]. Cells of the epicardium, endocardium and vascularized fibrotic tissue have been shown to play a critical role in this regenerative response through paracrine signals, such as FGF, TGF-β, IGF and retinoic acid [15–18]. The importance of systemic factors has only recently been addressed, focusing on the role of cardiac innervation [19]. To our knowledge, the potential impact of environmental or psychological factors has not yet been assessed in this context. In this study, we aimed to determine if the efficient regenerative mechanism of the zebrafish heart could be challenged by stress stimuli, in particular by intermittent exposure to crowding.

Our work revealed that under stress, the robustness of heart regeneration in zebrafish is compromised, resulting in persisting fibrosis at the site of cryoinjury. We show that systemic stress hormones play a major role in this context, particularly by acting on CM proliferation via secondary downstream pathways, such as the IGF axis. The modulation of the stress response with anxiolytic drugs partially rescued the regenerative impairment in the stressed animals. For the first time, we integrate cardiac-exogenous components in the tight regulation of heart regeneration in zebrafish.

## 2. Results

### 2.1. Diverse stressors induce cortisol-mediated signalling in adult zebrafish

**Figure 1.**
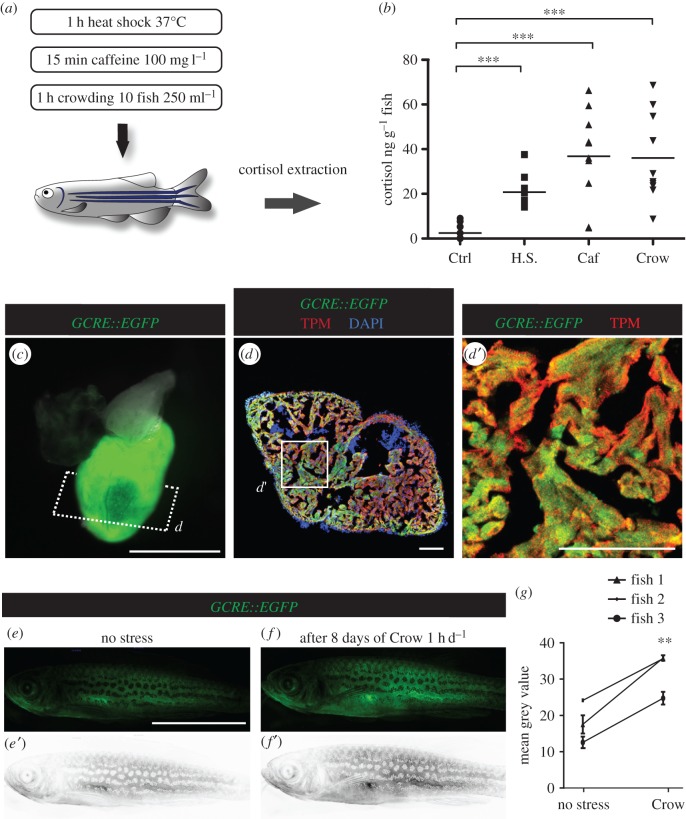
In adult zebrafish, stress stimulates cortisol secretion and activates GCR signalling. (*a*) Adult zebrafish were exposed to different stressors. (*b*) Whole-body cortisol levels are shown as a dot plot. Each dot represents the value obtained for one fish. The dash indicates the mean value of each group (*N* ≥ 10 fish; ***p < 0.001). H.S., heat shock; Caf, caffeine, Crow, crowding. (*c*) Live heart dissected from *Tg*(*GCRE::EGFP*) fish display GFP in the ventricle. (*d*,*d*′) Immunofluorescence analysis of *Tg*(*GCRE::EGFP*) heart section demonstrates a colocalization between tropomyosin and GFP. However, this transgenic reporter is not ubiquitously expressed in adult tissues. (*e*,*f*) Fluorescent images of the same transgenic animal *Tg*(*GCRE::EGFP*) reveal an enhanced glucocorticoid receptor (GCR) transcriptional activity in the whole body at 8 days after 1 h of daily crowding, as compared to the state before stress. (*e*′,*f*′) Converted images of the GFP signal into a grey scale using Image J software for quantification of fluorescence. (*g*) Graph showing the mean converted fluorescence values obtained for three different fish before and after the period with daily acute stress exposure. **p < 0.01. Scale bars: (*c*) 1 mm, (*d*) 100 µm and (*e*) 10 mm.

Like in humans, the stress level in zebrafish can be assessed by measuring the concentration of the primary stress hormone cortisol in the body fluids [3–5,20]. To test diverse stressing conditions of physical, chemical and psychological nature, we exposed the adult zebrafish to 1 h of heat shock (37°C; 10 fish l^−1^), 15 min of caffeine (100 mg l^−1^; 10 fish l^−1^) and 1 h of crowding (10 fish 250 ml^−1^). We found that a single stress exposure resulted in a 10- to 40-fold increase in the mean cortisol concentrations of stressed fish as compared to control fish (figure 1*a*,*b*). Interestingly, crowding robustly elevated the cortisol levels, suggesting a strong sensitivity of the zebrafish to a psychological stress, which is consistent with other aquatic biology studies [21]. We considered crowding as a particularly suitable experimental approach to study the effect of perceived stress on heart regeneration in zebrafish.

Cortisol is a natural lipophilic glucocorticoid hormone, which affects gene transcription mainly by modulating the nuclear glucocorticoid receptor (GCR) activity [22,23]. The GCR activity can be monitored in the transgenic fish *Tg*(*9* *×* *GCRE-HSV.Ul23:EGFP*)*ia20*, referred to as *GCRE::EGFP*, which expresses fluorescent proteins under the control of transcriptional GCR-responsive elements [24]. This transgene is expressed in various body parts, including the ventricle, as shown in dissected whole heart and immunostained sections (figure 1*c*,*d*). The live-imaging of the same transgenic animals before and after daily crowding for 1 h during 7 days (d) revealed an upregulation of *GCRE::EGFP* expression in the whole body (figure 1*e*–*g*). A basic level of glucocorticoid signalling is essential for the regulation of a broad range of cellular responses during homeostasis [22]. Analysis of the GCRE-GFP expression in uninjured zebrafish heart allows detecting tissues or cells that are responsive to glucocorticoids. Although we observed inter-individual variability in the baseline activation of this pathway (intensity of GFP signals), the exposure to acute stress was sufficient to elevate the transcriptional activity of GCR signalling in the same animal.

### 2.2. Daily stress after cryoinjury blocks cardiac regeneration

**Figure 2.**
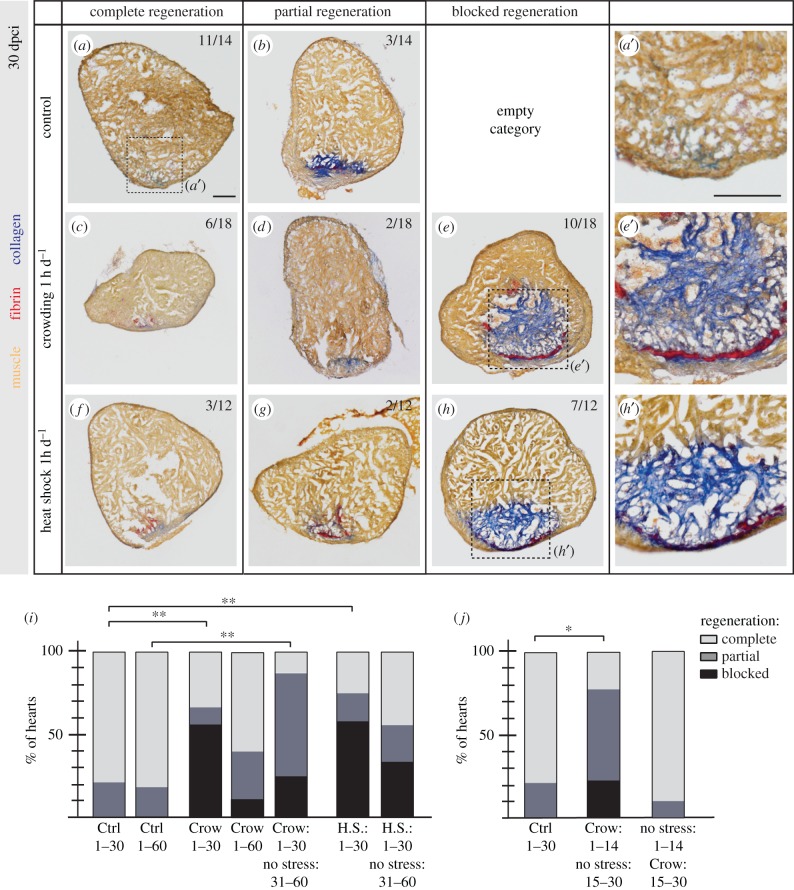
Daily stress blocks cardiac regeneration after cryoinjury. (*a*–*h*) Heart sections at 30 dpci after AFOG staining showing the myocardium (orange), fibrin (red) and collagen (blue). The number in the upper right corner of each image represents the fraction of the analysed fish with the displayed phenotype. (*i*,*j*) Histograms represent the percentage of zebrafish hearts with complete (white), partial (grey) or blocked (black) regeneration. Numbers indicate days after cryoinjury for each experimental group. The regeneration scores were evaluated according to the area of collagen on multiple sections per each heart. *N* ≥ 8; *p < 0.05, **p < 0.01, Fisher's exact test. Scale bars, 100 µm.

To study the impact of repetitive acute stress on heart regeneration, we exposed fish to real and perceived stressors, namely heat shock and crowding, for 1 h d^−1^, during 30 days after ventricular cryoinjury (dpci). One month is sufficient to replace the transient fibrosis after cryoinjury and to regenerate the damaged organ in 80% of non-stressed animals, as visualized by AFOG (Aniline blue, acid Fuchsin, Orange-G) staining (figure 2*a*,*i*) [25]. In contrast with controls, the majority of stressed animals failed to efficiently regenerate the heart, as collagenous tissue was persisting in the injured area. Specifically, only 33% of animals regenerated the heart when exposed to daily crowding and only 25% after heat shock (figure 2*c*–*i*). These results demonstrate a detrimental impact of stress on heart regeneration.

Chronic stress is known to affect metabolism and feeding behaviour [26]. To uncouple a stressful experience from nutrition, the animals were fed twice daily approximately 2 h before and 6 h after stress exposure. In this regard, body weight measurement after cryoinjury and at 30 dpci revealed no impact of stress on body weight, indicating normal food intake (electronic supplementary material, figure S1Q). Interestingly, we observed that control animals showed enhanced blood glucose levels at the time of completion of heart regeneration at 30 dpci, while the stressed animal values remained similar to those at 7 dpci (electronic supplementary material, figure S1R).

To determine whether stress irreversibly blocks regeneration or delays its efficiency, we extended the duration of the regenerative process until 60 dpci. At this time point, a similar proportion of control animals achieved complete regeneration as compared to 30 dpci (figure 2*i*, electronic supplementary material, figure S1*a* and *b*). Animals that were stressed by crowding displayed an improved regenerative outcome at 60 dpci as compared to 30 dpci; however, they did not reach the efficient regeneration of the controls (figure 2*i*; electronic supplementary material, figure S1*c*–*e*). We concluded that psychological stress impairs regeneration mostly during the first four weeks, with a progressive attenuation of its effect in the long term. To test this hypothesis, we designed an experiment in which the animals were stressed for 30 days either by crowding or heat shock, followed by a recovery period in normal conditions for an additional 30 days. Interestingly, the regenerative outcome was similar between the groups exposed to crowding during 60 days or only during the initial 30 days followed by an additional month at normal conditions (figure 2*i*; electronic supplementary material, figure S1*f*–*k*). This indicates that the initial period of stress has a more potent effect on heart regeneration, and the prolonged stress progressively loses its detrimental impact, probably due to the habituation of the animals to regular averse stimuli.

The process of zebrafish heart regeneration after cryoinjury involves the initial phase of wound healing and cardiac blastema formation in the course of 14 days after injury, followed by the progressive replacement of fibrotic tissue with a new myocardium during the two subsequent weeks [14]. To determine which of these cellular events is more susceptible to stress, we performed an experiment with two groups of zebrafish stressed either during the first (1–14 dpci) or during the second phase (15–30 dpci) of cardiac regeneration. Our results demonstrated that daily exposure to crowding during the initial period impaired heart regeneration in 78% of animals, while stress exposure during the progressive phase did not cause any phenotype in comparison to controls (figure 2*j*; electronic supplementary material, figure S1*l*–*p*). This reveals a stronger repercussion of stress on the first phase of cardiac regeneration that is characterized by initiation of the regenerative programme.

**Figure 3.**
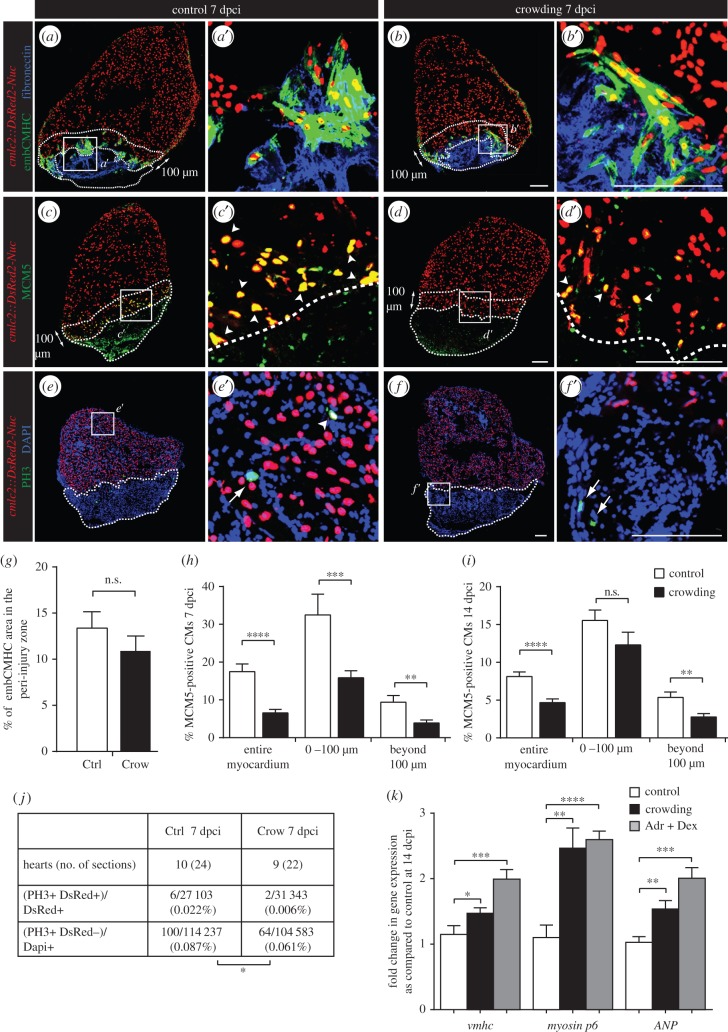
Daily acute stress exposure after cryoinjury affects CM proliferation and triggers compensatory mechanisms associated with cardiac overload. (*a*–*f*) Immunofluorescence staining of heart sections at 7 dpci of *cmlc2::DsRed2-Nuc* (cardiac nuclei, red) transgenic zebrafish of control and stressed animals. Dashed lines encircle the cryoinjured parts and a 100 µm-wide margin of the remaining myocardium along the injury border (peri-injury zone). (*a*–*b*′) No change in expression of embCMHC (embryonic isoform of cardiac myosin heavy chain, MAb N2.261, green) and fibronectin (blue) is observed between both experimental groups, *N* ≥ 4. (*c*–*d*′) The G1/S phase marker MCM5 (green) is reduced in CM nuclei of the stressed animals, particularly in the peri-injury zone. Arrowheads indicate double positive cells. (*e*–*f*′) The mitosis marker PH3 (green) is less frequent in the stressed animals as compared to controls. Arrows indicate mitotic non-CMs. (*g*–*j*) Quantification of immunofluorescence analysis shown in the representative images. *N* ≥ 8 hearts; three sections per heart. (*k*) qRT-PCR experiments revealed that exposure to crowding or dexamethasone/adrenaline (Adr + Dex) for 1 h d^−1^ during 14 days after cryoinjury results in upregulation of genes associated with cardiac compensatory response; *N* ≥ 2 sets, 10 hearts each. *p < 0.05, **p < 0.01, ***p < 0.001, ****p < 0.0001. Scale bars, 100 µm.

The stimulation of the regenerative programme involves an activation of developmental genes in the myocardial zone adjacent to injury [11,27]. Accordingly, we analysed the expression of the embryonic cardiac myosin heavy chain (embCMHC) using monoclonal antibody N2.261 [14]. At 7 dpci, both control and stressed fish displayed a similar N2.261 immunoreactivity at the peri-injury zone of the myocardium (figure 3*a*,*b*,*g*). Thus, the regenerative failure of stressed animals is not associated with defective re-expression of embryo-specific sarcomeric proteins.

To identify the mechanisms responsible for the impaired regeneration after crowding, we analysed CM proliferation, which is an essential cardiac regenerative process [12,13,15,28]. We selected two cell cycle markers, MCM5 (minichromosome maintenance complex component 5; a protein of G1/S phase) and PH3 (phospho-Ser10-histone H3; a protein of mitotic, condensed chromosomes) [29], in the *cmlc2::DsRed2-Nuc* transgenic strain, in which the cardiac nuclei are labelled with DsRed (figure 3*c*–*f*). The quantification of MCM5-positive and DsRed-positive nuclei revealed a twofold reduction in the proportion of proliferating CMs in crowding conditions at 7 and 14 dpci (figure 3*h*,*i*). Importantly, quantification of PH3-positive nuclei demonstrated a decrease of mitotic events in cardiac cells of the stressed animals (figure 3*e*,*f*,*j*). We concluded that crowding globally suppresses cardiac proliferation within the entire myocardium, which might account for impaired heart regeneration.

In zebrafish, the mature adult CMs maintain their proliferative capacity during the ontogenic growth throughout the lifespan of the animal. To test whether stress impairs cardiac cell proliferation not only during regeneration but also during normal growth, we used the juvenile fish model system [30]. In our experiment, two month old animals were transferred from standard to low-density condition for 10 days to stimulate rapid growth, and twice daily were exposed to 1 h of crowding (electronic supplementary material, figure S2). After such a transfer, control animals contained an elevated number of MCM5-positive cardiac cells in comparison to animals at standard conditions. By contrast, acute exposure to crowding prevented stimulation of cardiac cell proliferation despite the maintenance for 22/24 h at low-density conditions (electronic supplementary material, figure S2*a*–*c*). Moreover, weight analysis of the animals revealed that daily exposure to stress suppresses the weight gain induced by the transfer to low-density conditions (electronic supplementary material, figure S2*d*). Thus, stress not only interferes with heart regeneration in adult fish but also affects the organismic and cardiac growth of the juvenile animal.

### 2.3. Deregulation of cortisol levels is the principal hormonal effector of the stress-induced regenerative impairment

**Figure 4.**
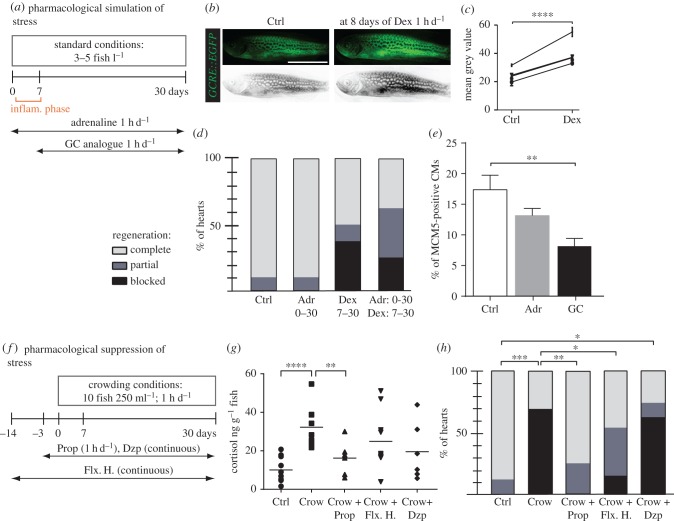
Pharmacological simulation of stress reproduces the crowding phenotype, which can be partially rescued by treatment with propranolol and fluoxetine hydrochloride. (*a*,*f*) Experimental design. (*b*) Live *Tg*(*GCRE::EGFP*) fish imaged for fluorescence (green converted to grey) before (Ctrl) and after 8 days of 2 mg l^−1^ dexamethasone (Dex) treatment for 1 h d^−1^ validates the activity of the drug. (*c*) Quantification of grey-converted fluorescence shown in (*b*); *N* = 4. (*d*,*h*) Regenerative scores of AFOG stained hearts at 30 dpci; *N* ≥ 8. (*e*) Quantification of MCM5 and DsRed-positive nuclei at 7 dpci in control and after treatments; *N* ≥ 6. (*g*) Measurements of whole-body cortisol after single stress exposure in fish with different pretreatment; *N* ≥ 5. Drugs: glucocorticoid (GC), dexamethasone (Dex, 2 mg l^−1^, 1 h d^−1^), adrenaline (Adr, 1 mg l^−1^, 1 h d^−1^), hydrocortisone (1 mg l^−1^), propranolol (Prop, 1 mg l^−1^, 1 h d^−1^), fluoxetine hydrochloride (Flx. H., 100 µg l^−1^, continuous), diazepam (Dzp, 1 mg l^−1^, continuous treatment). *p < 0.05, **p < 0.01, ***p < 0.001, ****p < 0.0001. (*b*) Scale bar, 10 mm.

As in humans, the stress response system in zebrafish is mainly driven by the activation of the hypothalamus–pituitary axis and the sympathetic nervous system leading to the production and release of cortisol and nor-/adrenaline [3,4]. To pharmacologically mimic the stress, we selected a synthetic glucocorticoid, dexamethasone (Dex), together with adrenaline for acute treatments (1 h d^−1^; figure 4*a*). To verify the pharmacological activity of Dex, we analysed its effect on the transcriptional activation of the glucocorticoid transgenic reporter *GCRE::EGFP*. Monitoring of the transgenic fish exposed to Dex during 7–9 days revealed an upregulation of *GCRE::EGFP* expression in the whole body (figure 4*b*,*c*). This result confirms that the exogenous Dex stimulates the systemic activation of the glucocorticoid-signalling pathway.

To test the effect of pharmacological stress on heart regeneration, fish were treated during 30 dpci with Dex (2 mg l^−1^, 1 h d^−1^), adrenaline (1 mg l^−1^, 1 h d^−1^), or a mixture of Dex and adrenaline. As the glucocorticoids are known to interfere with the immune and wound healing response, we started the treatment on day 7 after cryoinjury, to allow normal initial repair of the injured heart (figure 4*a*). To address the potential effect of this acute treatment on inflammation, we performed immunostaining against L-plastin, a leucocyte-specific actin-bundling protein, at 30 dpci [31]. This experiment revealed no significant impact on leucocyte recruitment (electronic supplementary material, figure S3*a*–*c*). The treatment with adrenaline did not affect heart regeneration at 30 dpci (figure 4*d*; electronic supplementary material, figure S3*d*–*g*). However, the daily 1 h treatment with Dex and Dex/adrenaline impaired regeneration in 50% and 62.5% of animals, respectively, which failed to complete the replacement of fibrotic tissue (figure 4*d*; electronic supplementary material, figure S3*h*–*m*).

To test the effect of glucocorticoids and adrenaline on CM proliferation, we performed drug treatments during 7 dpci. Quantification of MCM5-positive nuclei of CMs revealed that the administration of glucocorticoids elicited a significant reduction in CM proliferation, whereas adrenaline treatment did not have a significant effect (figure 4*e*; electronic supplementary material, figure S3*n*–*p*). Thus, enhanced glucocorticoid levels impair the proliferative capacity of adult zebrafish CMs.

To further characterize the response of cardiac cells to stress stimuli, we examined the expression level of several sarcomeric genes, such as *ventricular myosin heavy chain* (*vmhc*) and *myosin p6,* as well as a mammalian marker of cardiac overload, *atrial natriuretic factor* (*ANF*). Quantitative real-time PCR performed at 14 dpci revealed that the hearts exposed to perceived or pharmacological stress showed a significant increase in the expression level of these genes (figure 3*k*). Thus, the stress-induced decrease in CM proliferation is accompanied by a compensatory response of cardiac cells to increased haemodynamic load.

### 2.4. Treatment with non-selective β-blocker and serotonin-reuptake inhibitor partially rescued the stress-induced cardiac regenerative defect

Fine-tuned levels of glucocorticoids maintain the principal metabolic functions that are essential for life [22]. Therefore, it is expected that the complete suppression of glucocorticoid production or activity could lead to detrimental effects on the global health of the animals, which would not be appropriate in the study of psychological stress on cardiac regeneration. Indeed, we observed that an anti-glucocorticoid drug, mifepristone (RU-486), a synthetic steroid antagonist of a glucocorticoid receptor in target tissues [32,33], had toxic effects after exposure even at low concentration for 7 days (data not shown). We anticipated that a more specific approach for attenuation of stress without global side effects would be based on treatment with antidepressants and anxiolytics that have been approved for human therapy. Accordingly, to rescue cardiac regeneration in the stressed zebrafish, we selected several commonly known drugs, such as propranolol (β-blocker, a non-selective β-adrenoreceptor antagonist) [34,35], fluoxetine hydrochloride (Prozac^®^, a serotonin-reuptake inhibitor) and diazepam (Valium^®^, a benzodiazepine drug which enhances GABA neurotransmitter effect) (figure 4*f*). First, we validated the inhibitory effects of the selected drugs on the stress response in zebrafish using the cortisol assay (figure 4*g*). Then, we evaluated the potential capacity of these drugs to counteract the negative effect of stress on heart regeneration at 30 dpci (figure 4*h*). We found that the administration of diazepam did not rescue the stress-induced regenerative impairment (figure 4*h*; electronic supplementary material, figure S4*e*–*g*). However, propranolol and fluoxetine hydrochloride were beneficial for cardiac regeneration in the stressed fish (figure 4*h*; electronic supplementary material, figure S4*h*–*l*). Specifically, the exposure to propranolol nearly completely suppressed the detrimental effect of crowding, resulting in the regenerative outcome similar to control. After the treatment with fluoxetine hydrochloride, only 20% of fish displayed a severe lack of regeneration, as compared to 60% in the stressed animals. The different effects of diazepam, propranolol and fluoxetine hydrochloride could be explained by their distinct pharmacological actions. Indeed, diazepam, as opposed to propranolol and fluoxetine hydrochloride, enhances the inhibitory GABA activity and reduces the excitability of cells [36]. We concluded that anxiolytics might decrease the general alertness state of the fish, and consequentially favour heart regeneration in the crowding conditions (electronic supplementary material, figure S5).

### 2.5. Whole transcriptome analysis revealed significant alterations in gene expression in the stressed animals

Heart regeneration in zebrafish involves a tight communication between different cell types, which is mediated by secreted proteins. Accordingly, we analysed the expression of several cell-signalling proteins that are known to be upregulated in non-cardiac tissues of the regenerating hearts. Immunofluorescence analysis did not reveal effects of stress on the expression of retinal aldehyde dehydrogenase 2 (Raldh2, Aldh1a2), a retinoic acid-synthesizing enzyme that is induced in the epi/endocardium after cardiac injury [16] (electronic supplementary material, figure S6*a*,*b*). Similarly, we did not observe a difference in expression of fibronectin and tenascin C, which are extracellular matrix proteins of the transient fibrotic tissue (figure 3*a*,*b*; electronic supplementary material, figure S6*c*,*d*). To test whether apoptosis could account for the impaired connective tissue resorption, we performed TUNEL assay. We did not observe a significant change in the number of apoptotic cells (electronic supplementary material, figure S6*e*–*g*).

To identify genes by which the stress could influence cardiac regeneration after cryoinjury, we performed two independent whole transcriptome analyses at 14 dpci, a critical period where the transition between the transient scar formation and resorption is typically detectable. The first RNA-sequencing experiment (RNA-seq1) was done with the cryoinjured parts of the ventricles, whereas the second analysis was carried out with the whole ventricles (RNA-seq2) (electronic supplementary material, figure S7*a*–*d*). Although the principal component (PC) analysis (PCA) shows that two independent experiments (RNA-seq1 and RNA-seq2) can be distinguished in the function of PC1, the second dimension (PC2) demonstrates that we can discriminate between the stressed and control samples in both experiments (electronic supplementary material, figure S7*e*). This increases the robustness of our data as we could differentiate the stressed and control samples in two individual experiments. Each experiment was first analysed independently before combining both with DESeq2 [37]. The fusion of both analyses (fused RNA-seq1 and RNA-seq2) led to a list of 1576 differentially expressed (DE) genes with adj-*p*-value < 0.05.

The use of Pathway Studio^®^ enabled us to make different gene enrichment analyses with the corresponding human orthologous genes. The most significant gene sets are listed in the electronic supplementary material, table S1 and S2. Of particular relevance were the changes observed in the gene cluster for the TGF-β signalling pathway, extracellular matrix turnover and in cell cycle regulation. Interestingly, we found that a large portion of the zebrafish genes, which were significantly changed between the stressed and control fish and did not have human orthologous genes, correspond to non-coding RNAs (electronic supplementary material, table S3). Further analysis of the implication of these molecular pathways in the stress-induced cardiac regenerative failure would be of particular interest.

**Figure 5.**
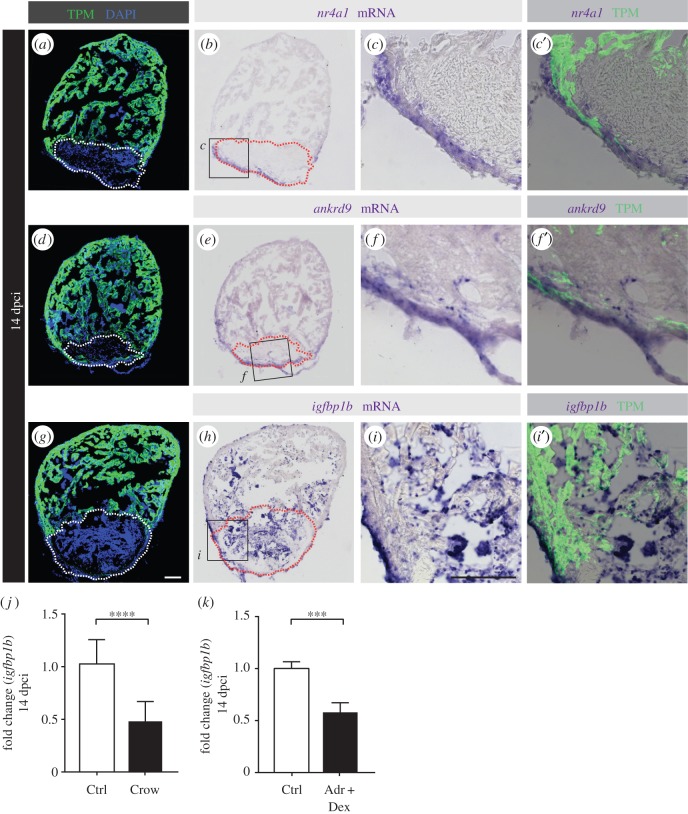
RNA-seq analyses of control versus stressed animals identified three candidate genes, *nr4a1, ankrd9* and *igfbp1b*, which are expressed in non-CMs of cryoinjured hearts. (*a*,*d*,*g*) Sections of hearts at 14 dpci immunostained against tropomyosin (TPM, green) and DAPI (blue). (*b*,*e*,*h*) The same sections as in (*a*,*d*,*g*) after *in situ* hybridization with RNA antisense probes. (*c*,*f*,*i*) Higher magnification of the framed area shown in (*b*,*e*,*h*) overlapped with tropomyosin (TPM, *c*′,*f*′,*i*′) immunostaining. (*j*,*k*) qRT-PCR experiments showed that hearts at 14 dpci exposed to stress or dexamethasone/adrenaline treatment for 1 h d^−1^ display a decrease in *igfbp1b* expression (*N* ≥ 3 sets of 10 cryoinjured ventricles). ***p < 0.001, ****p < 0.0001. Dashed lines encircle the cryoinjured areas. Scale bars, 100 µm.

In a second step, we compared the results of each individual experiment with those of the fused RNA-seq1 and RNA-seq2. This analysis highlighted three genes, which were significantly downregulated by stress exposure, namely *ankrd9* (*ankyrin repeat domain 9*)*, nr4a1* (*nuclear receptor subfamily 4, group A, member 1*) and *igfbp1b* (*insulin-like growth factor binding protein 1b*)*.* We localized the expression of these mRNAs in the zebrafish heart after cryoinjury by *in situ* hybridization: *nr4a1* and *ankrd9* were located in the layer of non-CMs surrounding the cryoinjured area (figure 5*a*–*f*), whereas *igfbp1b* was predominantly detected in the cryoinjured part of the ventricle, possibly in connective tissue cells and blood cells, although some expression was also scattered throughout the endocardium (figure 5*g*–*i*). The absence of expression of these factors in CMs (labelled by tropomyosin immunostaining) implies a non-cell-autonomous effect of stress on cardiac regeneration and highlights the interdependence between different tissues to promote heart restoration after injury.

### 2.6. Stress decreases the activity of IGF signalling in the regenerating heart

Igfbp1b belongs to the protein superfamily that comprises carriers of insulin-like growth factors (IGF) and modulators of IGF availability and activity [38]. As IGF signalling plays a major role during fin regeneration and recently has been shown to be required for CM proliferation during zebrafish heart development and regeneration [18,39], we sought to further investigate the potential link between stress and IGF signalling. By means of quantitative real-time PCR performed at 14 dpci, we confirmed that the *igfbp1b* gene is downregulated in the hearts of stressed and stress hormone-treated animals (figure 5*j*,*k*).

**Figure 6.**
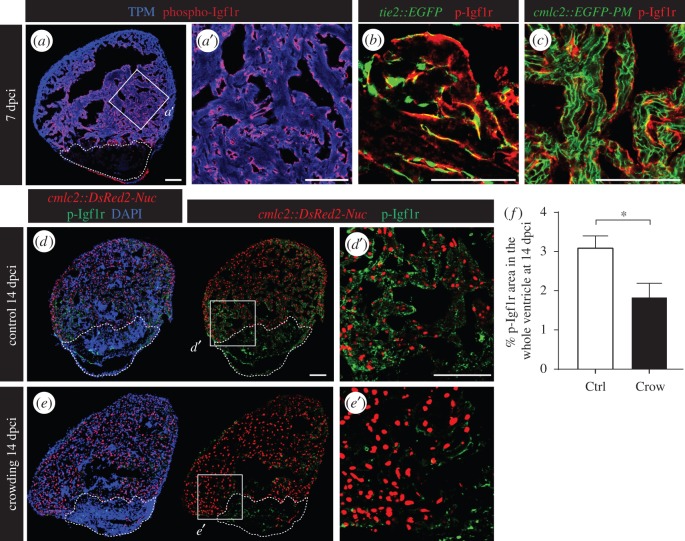
Daily stress decreases phospho-Igf1r activation in the regenerating heart. (*a*) Ventricular sections at 7 dpci reveal the presence of phospho-Igf1r (p-Igfr1, red) in the endocardium that covers cardiac muscle labelled by tropomyosin expression (TMP, blue). (*b*) p-Igf1r partially colocalizes with the pattern of endothelial cell layers demarcated by GFP expression in *tie2::EGFP* transgenic fish. (*c*) p-Igf1r does not overlap with the plasma membrane of CMs in *cmlc2::EGFP-PM* transgenic fish. (*d*,*e*) Sections of *cmlc2::DsRed2-Nuc* (red) hearts at 14 dpci display reduced p-Igf1r (green) expression in stressed animals. (*f*) Quantification of phospho-Igf1r area per ventricle at 14 dpci in control and stressed animals (*N* ≥ 7). *p < 0.05. Dashed lines encircle the cryoinjured areas. Scale bars, 100 µm.

To visualize the IGF signalling activity in the control hearts, we used antibody against a phosphorylated form of insulin-like growth factor receptor 1 (phospho-Igf1r) (figure 6*a*). The activated Igf1r was detected in the cells associated with the endocardium of the transgenic fish *tie2::EGFP* (figure 6*b*). We did not observe any co-localization between phospho-Igf1r and the plasma membrane of CMs in the transgenic fish *cmlc2::EGFP-PM* (figure 6*c*). The similarity between the *igfbp1b* expression pattern (figure 5*g*–*i*) and phospho-Igf1r immunodetection supports a possible modulatory interplay between both molecules. Indeed, similarly to *igfbp1b*, the level of phospho-Igf1r was significantly reduced in the stressed animals as compared to controls (figure 6*d*–*f*).

**Figure 7.**
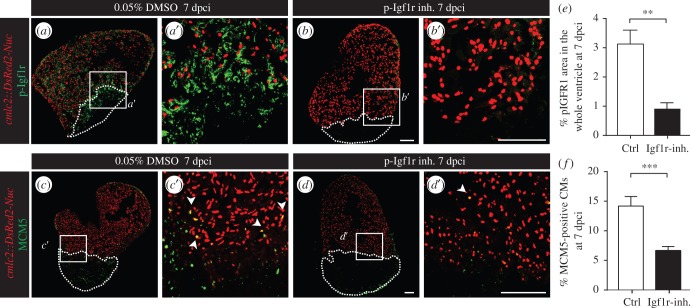
Inhibition of IGF signalling impairs CM proliferation. (*a*–*d*) Sections of *cmlc2::DsRed2-Nuc* (red) hearts at 7 dpci reveal that the treatment with 5 µM NVP-ADW742 (inhibitor of Igf1r) significantly reduces p-Igf1r levels (*a*,*b*, green) and MCM5-positive CMs (*c*,*d*, green). (*e*–*f*) Quantification of p-Igf1r (*e*, *N* ≥ 4) and the number of proliferating CMs (*f*, *N* ≥ 10) in the whole ventricles at 7 dpci. **p < 0.01, ***p < 0.001. Dashed lines encircle the cryoinjured areas. Scale bars, 100 µm.

To test whether the detrimental effect of stress is mediated through the downregulation of IGF signalling, we performed functional analysis of this signalling pathway after ventricular cryoinjury using NVP-ADW742, which was validated as a specific inhibitor of Igf1r kinase activity in the zebrafish fin [39]. At 7 dpci, treatment with 5 µM NVP-ADW742 markedly decreased phospho-Igf1r in the whole regenerating ventricle at 7 dpci, demonstrating the efficient inhibition of IGF signalling (figure 7*a*,*b*,*e*). Importantly, this phenotype was associated with decreased CM proliferation, suggesting a non-cell-autonomous effect of IGF signalling from the endocardium on CMs (figure 7*c*,*d*,*f*). Consistently, the inhibition of IGF signalling during 30 days after cryoinjury impaired regeneration (electronic supplementary material, figure S8). These results imply a potential causality link between the stress-mediated downregulation of *igfbp1b* expression, the reduction in IGF signalling and reduced CM proliferation resulting in impaired regeneration.

## 3. Discussion

The instinctive fight-or-flight reaction was originally developed to maximize the survival chance of an organism in life-threatening situations [6,7]. Our work demonstrates that real and perceived stress impairs the robust natural heart regeneration in zebrafish. This phenotype was associated with a reduced proliferative activity of CMs. Moreover, we found that repetitive stress exposure affects rapid cardiac growth in juvenile animals. Thus, the abnormal activation of the stress response appears to have global effects on the heart plasticity during adult regeneration and ontogenic growth. In the light of our results, particular attention has to be paid regarding the use of heat shock inducible zebrafish transgenic lines, which are frequently used in functional studies of heart regeneration.

To determine the molecular origin of these cellular effects, we performed two independent transcriptome analyses at 14 dpci, which commonly identified three differentially expressed genes: *ankrd9*, *nr4a1* and *igfbp1b*. We verified that all these genes were expressed in non-CMs of the cryoinjured ventricle. The *ankrd9* gene is poorly characterized in vertebrates, although its expression was decreased in a transcriptome analysis of chronic constant hypoxia in the zebrafish heart [40]. Nr4a1 (Nur77) belongs to the NR4A orphan nuclear receptors, which are hormone-sensitive transcription factors involved in the early response to pathological stimuli [41,42]. Interestingly, this receptor has already been implicated in mammalian liver regeneration [43,44] and is differentially regulated in the dorsal iris of the regenerating newt lens [45]. Thus, its potential role during heart regeneration has to be considered. Finally, Igfbp1b is known to act as a modulator of IGF signalling, and thus can influence different cellular processes such as proliferation or apoptosis [38,46].

**Figure 8.**
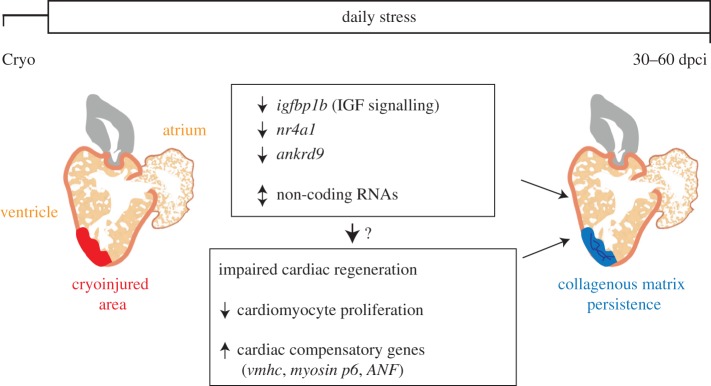
Stress suppresses CM proliferation resulting in impaired heart regeneration. Daily exposure to stress after cryoinjury leads to a delay in cardiac regeneration with scar persistence. Among the potential mechanisms underlying this negative effect, the stress-induced elevation of cortisol secretion appears to drive a shift from CM proliferation to other cardiac compensatory responses. In addition, daily acute stress is associated with significant changes in gene expression, such as the downregulation of *igfbp1b*, *nr4a1* and *ankrd9* in non-myocytes of the ventricle, indicating a non-cell-autonomous effect of stress on cardiac regeneration.

Among the identified genes, we focused on *igfbp1b*. Igfbps are important modulators of IGF availability and can have either stimulatory or inhibitory function dependent on the tissue and cellular context [47,48]. Our results pointed to a similar expression pattern of *igfbp1b* and phospho-Igf1r*.* Moreover, daily exposure to stress significantly reduced both *igfbp1b* expression and the activity of Igf1r. Similarly, Nakano *et al*. [8] have shown that acute physiological stress is associated with the downregulation of growth factor genes, such as IGF-1, in salmon. We showed that CM proliferation was significantly decreased in the zebrafish treated with a specific pharmacological inhibitor of Igf1r, a finding that is reminiscent of the observations made by Huang *et al*. [18]. One of the Igf1r ligands, Igf2, has been shown to be an important epicardial cell-derived mitogen, which stimulates CM proliferation during cardiac development [49]. Thus, we propose that the downregulation of *igfbp1b* and the consequent deregulation of IGF signalling is a possible mechanism that decreases CM proliferation, and subsequently leads to cardiac regenerative failure in the stressed animals (figure 8). Further studies are required to assess the precise role of Igfbp1b and IGF signalling during cardiac regeneration and to determine whether the activation of IGF signalling would be sufficient to rescue the detrimental effects of stress in the heart.

Various studies have already validated the usefulness of the zebrafish as a model in the stress research field due to high functional and organizational similarities with the human's sympathetic adrenal and HPA axes [3,4]. In our study, the acute administration of glucocorticoid reproduced the negative effects of stress on ventricular regeneration and CM proliferation, implying a causal relationship between the stress-associated disturbances in glucocorticoid signalling and the cardiac regenerative failure. In human chronic stress, the deregulation of the neuro-endocrine pathways is known to disfavour multiple physiological processes and can result in cardiovascular disorders, such as cardiac hypertrophy [22,50–54]. Consistently, we found that different genes associated with cardiac response to increased haemodynamic load were upregulated in the stressed zebrafish. However, the correlation between glucocorticoid level or activity and regeneration might certainly be more complex than simply linear. In this regard, additional experiments from our laboratory (data not shown) revealed that blocking cortisol secretion (CP154526, antagonist of corticotropin releasing hormone receptor) or activity (RU-486, glucocorticoid receptor antagonist) significantly affects cardiac regeneration and produced similar phenotype to the administration of glucocorticoids. A certain fine-tuned level of the stress hormones is as essential for regenerative mechanisms as it is to maintain the animal viable and healthy. A better understanding of the decisive mechanisms that favour either the cardio-protective or cardio-toxic effects of glucocorticoids would be of particular relevance for the therapeutic use of corticosteroids as well as for regenerative medicine.

In humans, the pharmacological strategy for decreasing the stress response is based on anxiolytics, which mainly act on the central nervous system by reducing the arousal state [34,55]. We found that the administration of fluoxetine hydrochloride and propranolol in the stressed zebrafish after cryoinjury significantly rescued heart regeneration. Consistently, Mahmoud *et al*. [19] have shown that, in contrast to cholinergic transmission inhibitors, the administration of propranolol after ventricular resection does not affect CM proliferation at 7 days post-amputation. Thus, acting on the central/peripheral nervous system to reduce the global alertness of the organism might re-establish the tightly regulated hormonal equilibrium, which is probably required for cardiac regeneration. Hence, it seems that environmental or psychological perturbations have a significant impact on the well-regulated machinery needed for ventricular restoration in zebrafish. Elucidating the regenerative processes that are particularly vulnerable to stress might provide new strategic perspectives to improve heart repair after myocardial infarction in humans.

## 4. Material and methods

### 4.1. Animal procedures

Our study includes wild-type fish AB (Oregon), *cmlc2::DsRed2-Nuc* [56], (*9* *×* *GCRE-HSV.Ul23:EGFP*)*ia20* [24], *cmlc2:eGFP-ras^s883^* [57] referred to as *cmlc2::EGFP-PM* (*plasma membrane*) and *tie2::EGFP* [58] zebrafish strains. Adult animals were 10–18 months old at 26°C. Cryoinjuries were performed as described previously [59].

#### 4.1.1. Assessment of cardiac regeneration

For the analysis of stress exposure or drug treatments on heart regeneration, the regenerative process was scored between 0 and 2.

*Score 0*: complete or almost complete cardiac regenerative process characterized by a replacement of the wound area with new myocardium and the absence of collagenous scar tissue.

*Score 1*: partial regeneration characterized by a new myocardium formation around the collagenous tissue of the postinfarcted area.

*Score 2*: blocked regeneration marked by the persistence of fibrotic tissue and lack of new muscle formation along the myocardial wall.

#### 4.1.2. Pharmacological simulation of stress response

To mimic the effect of stress on heart regeneration, the zebrafish were treated with hydrocortisone (hydrocortisone 21-hemisuccinate sodium salt, Sigma, H2270, 1 mg l^−1^, diluted in system water, treatment changed every day), dexamethasone (Sigma, D1756, dissolved in DMSO at the 20 mg ml^−1^ stock concentration and added in fish water for a final concentration of 2 mg l^−1^, 1 h d^−1^) and/or adrenaline (adrenaline amino 1 mg, amino Ag, 1 mg l^−1^, 1 h d^−1^).

#### 4.1.3. Pharmacological suppression of stress response

For the stress-rescue experiments, the following drugs were used: mifepristone (RU-486, Sigma, M8046, dissolved in DMSO at the 25 mg ml^−1^ stock concentration and added in fish water for a final concentration of 2.5 mg l^−1^, 1 h d^−1^), propranolol (Prop, Sigma, P0884, dissolved in ddH_2_O at the 10 mg ml^−1^ stock concentration and added in fish water for a final concentration of 1 mg l^−1^, 1 h d^−1^, 3 days pretreatment), fluoxetine hydrochloride (Flx. H., TOCRIS, 0927, dissolved in DMSO at the 1 mg ml^−1^ stock concentration and added in fish water for a final concentration of 0.1 mg l^−1^, continuous treatment, 2 weeks pretreatment) and diazepam (Dzp, Valium®, Roche (injection solution), 1 mg l^−1^, continuous treatment, 3 days pretreatment). In the case of continuous treatment, the drug was replaced every 3 days. For acute treatments, the zebrafish were treated with the corresponding drugs in the morning, before the stress exposure.

#### 4.1.4. Pharmacological inhibition of IGF signalling

The Igf1r inhibitor NVP-ADW742 (Novartis Pharma AG) was dissolved in DMSO at 10 mM stock concentration and was added in fish water for a final concentration of 5 µM.

### 4.2. Histology, immunohistochemistry and *in situ* hybridization

The hearts were collected and fixed overnight at 4°C in 2% paraformaldehyde. Then, they were rinsed in PBS and equilibrated in 30% sucrose before embedding in OCT and cryosectioning at a thickness of 16 µm. Aniline blue, acid Fuchsin, Orange-G (AFOG) staining, TUNEL assay, *in situ* hybridization and immunohistochemistry were performed on heart sections as previously described [25]. To perform the different immunostaining shown in this study, the following primary antibodies were used: sheep anti-digoxigenin-fluorescein at 1 : 500 (Roche, 11 207 741 910), rabbit anti-glucocorticoid receptor at 1 : 200 (GeneTex, GTX101121), rabbit anti-DsRed (Clonetech, 632 496) at 1 : 200, rabbit anti-fibronectin at 1 : 400 (Sigma-Aldrich, F3648), chicken anti-L-plastin at 1 : 500 (kindly provided by P. Martin, Bristol), rabbit anti-MCM5 at 1 : 5000 (provided by Soojin Ryu), mouse anti-N2.261 (embCMHC, developed by H.M. Blau, obtained from Developmental Studies Hybridoma Bank), mouse anti-*p*-histone 3 at 1 : 200 (Clone 3H10, Millipore), rabbit anti-phospho-Igf1r (Tyr 1161) at 1 : 50 (Santa Cruz), rabbit anti-Raldh2 at 1 : 400 (GeneTex, GTX124302), rabbit anti-tenascin C at 1 : 500 (USBiological, T2550-23), mouse anti-tropomyosin at 1 : 100 (developed by J. Jung-Chin Lin and obtained from Developmental Studies Hybridoma Bank, CH1). The secondary antibodies (diluted 1 : 500) were: goat anti-rabbit Alexa Fluor 488, goat anti-mouse Alexa Fluor 488 (Molecular Probes), goat anti-rabbit Cy3 or Cy5. DAPI was used at a 1 : 2000 dilution and phalloidin (F-actin labelling) at 1 : 500. For phospho-Igf1r immunofluorescence, a Tyramide Signal Amplification kit (TSA Plus Cyanine 5 System) was used after incubation with anti-rabbit IgG (light chain) horseradish peroxidase (Jackson ImmunoResearch, 1 : 500). After antibody staining, cardiac tissue imaging was performed at different 20× and 63×magnifications with confocal microscopes (Leica TCS-SP5 and Leica TCS-SPE-II). ImageJ software was used to perform the subsequent image analysis. To enhance the visibility of the blue fluorescent colour on the final figures, we modified the colour profile with Adobe Photoshop using CMYK conversion. In the figures, the heart sections are displayed with the injured zone oriented to the bottom of the image.

#### 4.2.1. *In situ* hybridization

Several digoxigenin-labelled RNA antisense probes were generated by PCR amplification of specific cardiac cDNA sequences. The forward (F) and reverse (R) primers are listed in table 1. The reverse primers were synthesized with the addition of T3 polymerase.

**Table 1.** Primers for qRT-PCR and *in situ* hybridization. To generate antisense probes, the reverse primers were synthesized with addition of the promoter for T3 polymerase.

gene	gene ID	forward primer (5′>3′)	reverse primer (5′>3′)	product (bp)
qRT-PCR				
*β-actin*	ENSDARG00000037870	TTGGCAATGAGAGGTTCAGG	TGGAGTTGAAGGTGGTCTCG	55
*vmhc*	ENSDARG00000079564	CCT GCG AAA GTC AGA TCG AG	CTG GCT CAT GAA GGA AGG TG	214
*myosin p6*	ENSDARG00000090637	AAGAGCTGGCCAATGCAAAC	GGCACGCAGTTTATTCACTTG	110
*igfbp1b*	ENSDARG00000038666	TGT GGA GCA CCA CCC TAC TG	GGA TGG CGT TGA GTT GTG AC	106
antisense probe for *in situ* hybridization				
*igfbp1b*	ENSDARG00000038666	GGAGCACCACCCTACTGAAG	TGTTGAGGTCTAGCGTGAGG	804
*igfbp1b*	ENSDARG00000038666	GTGTGTGTTGGATGCGTCTC	CAGTGTGTGAGCTCCTGTGG	690
*nr4a1*	ENSDARG00000000796	GAGCTCCCTCTTCAGCTCAG	TTTGCATCCTTCACAAGTGC	739
*nr4a1*	ENSDARG00000000796	TCCCTACCCAGCATCACTTC	ACAGCTTTGGGTTTGGATG	849
*ankrd9*	ENSDARG00000028804	GGTGTTTCATTGGGAAGACG	GGCCTGATCGAGTCTGAAG	722
*vmhc*	ENSDARG00000079564	CTGCACTCCCAGAACACAAG	TCAACCAGATCTTGCAGACG	461

After hybridization, the probes were detected by the use of anti-digoxigenin AP-conjugated antibody (Dig labelling system, Roche).

### 4.3. Measure of stress level

#### 4.3.1. Cortisol extraction

Stress level in zebrafish was quantified by performing cortisol extractions as previously described [20]. Briefly, the zebrafish were frozen in liquid nitrogen immediately after the stress exposure. This procedure was performed in the morning as the secretion of cortisol follows a circadian rhythm. After euthanasia, each fish was weighed and cut into small pieces before homogenization in 500 µl of ice-cold PBS for 1 min. The rotor blade was washed in an additional volume of 500 µl PBS. The homogenates were transferred into glass tubes and the organic fraction containing cortisol was extracted from the rest by the addition of 5 ml diethyl ether, vortexing and centrifugation at 7000*g* for 10 min. The organic layer containing cortisol (top yellowish layer) was transferred to a fresh glass tube. The extraction procedure was repeated three times. To allow ether evaporation, the collected cortisol samples were maintained overnight under the hood before reconstitution in 1 ml 1× PBS. The quantification of cortisol level in each sample was performed with the use of a salivary cortisol enzyme immunoassay kit (Salimetrics, 1-3102-5) according to the manufacturer's instructions. The cortisol concentrations were normalized based on the body weight of the zebrafish and described as ng g^−1^ body weight.

#### 4.3.2. Monitoring of *Tg(9 × GCRE-HSV.Ul23:EGFP)ia20* transgenic zebrafish

The GCR activity was monitored in transgenic fish *Tg(9 × GCRE-HSV.Ul23:EGFP)ia20*, referred to as *GCRE::EGFP*. For each analysis, the same fish was imaged on three successive days before and after the period of stress at 6, 7 and 8 days of acute crowding. The pictures were always taken at the same time of the day to cope with circadian changes in GCR activity. The quantification of the GFP signal was performed after the conversion of the image into a grey scale using ImageJ software.

### 4.4. Glucose measurements

Glucose measurements were performed as previously described [60]. Briefly, fish were anaesthetized and euthanized in ice-cold facility water. An incision was performed between the anal fin and the caudal fin to induce bleeding [61]. Blood glucose levels were determined by using the FreeStyle Freedom Lite, 0.3 µl sample (Abbott) glucose meter and test strips.

### 4.5. RNA-seq analysis

Two whole transcriptome analyses were performed. For the first analysis (RNA-seq1), 1 × 15 cryoinjured parts were used for the control and stress groups. The use of *cmlc2:eGFP-rass883* (referred to as *cmlc2::EGFP-PM*) transgenic zebrafish, expressing GFP in the plasma membrane of CMs, enabled us to identify and isolate the cryoinjured parts of the ventricle. The second analysis was performed with 2 × 10 whole ventricles per group (RNA-seq2). After homogenization with a Tissue Lyser LT, the samples were transferred in MaXtract High Density tubes (Qiagen, cat.no 129056) before the addition of GenElute-LPA (Sigma, cat.no 56575), a synthetic polymer, which acts as a carrier for DNA/RNA. RNA was then isolated according to the Trizol reagent manual (Life Technologies, cat. no. 15596-026) and purified according to the RNeasy MinElute Cleanup Kit manual (Qiagen, cat. no 74004).

RNA libraries were prepared according to the TruSeq RNA Sample Preparation v2 Guide (Illumina) and 100 bp single-ends RNA sequencing was performed with an Illumina HiSeq2500 machine. The RNA-seq libraries consisted of 48 mio–67 mio reads for the first analysis (RNA-seq1) and of 2.4 mio–3.5 mio reads of 100 bp in length for the second analysis (RNA-seq2). Reads were first checked for sequence quality (FastQC, www.bioinformatics.babraham.ac.uk/projects/fastqc). The last 20 bp of the libraries were trimmed due to bad quality. The libraries were then mapped to the zebrafish genome (v. 9) using the spliced mapping approach implemented in TopHat2 [62]. For each gene (annotation version 9.75) the number of reads mapping to it were counted using the program HTSeq-count (www.w-huber.embl.de/users/anders/HTSeq v. 0.5.4p3). To test for significant differences of the expression levels between treatments the R-package DESeq [63] was used. *p*-Values were corrected for multiple testing following Benjamini & Hochberg [64].

#### 4.5.1. RNA-seq analysis

Two RNA-seq experiments (RNA-seq1 (one replicate, 15 cryoinjured parts per group) and RNA-seq2 (two replicates, 10 cryoinjured ventricles per group) were analysed using classical pipelines: QC with Fastqc (http://www.bioinformatics.bbsrc.ac.uk/projects/fastqc), mapping with TopHat2 [62], counting with HTSeq-count [65] and statistical analysis with DESeq1 [63]. Both analyses led to DE gene lists (28 for RNA-seq1, 115 for RNA-seq2 and with adj-*p*-value < 0.05).

As DESeq1 is very conservative, we reanalysed both experiments together (as we would have triplicates) with DESeq2 [37]. This analysis (fused RNA-seq1 and RNA-seq2) led to a list of 1576 DE genes with adj-*p*-value < 0.05. Although the PCA shows that the two experiments (RNA-seq1 and RNA-seq2) could be easily distinguished (PC1), the second dimension (PC2) allowed us to discriminate between the stressed versus control samples in both experiments.

#### 4.5.2. Pathway analysis

We performed a pathway analysis with Pathway Studio (Pathway Studio v. 9.0, Aridane Genomics, Rockville, MD, USA). Using this tool, we were able to highlight several pathways and regulatory networks potentially involved in cardiac regeneration.

### 4.6. Quantitative real-time PCR

RNA was extracted according to the Trizol reagent manual (Life Technologies) with the use of MaXtract High Density tubes. cDNA was synthesized with the Super-Script-II Reverse-Transcriptase (Invitrogen) using 1.5 µg of RNA. The primers used for different amplifications are listed in table 1.

### 4.7. Statistical analysis

All results are expressed as the mean ± s.e.m.. Statistical analyses have been performed with GraphPad Prism.

## Supplementary Material

Supplementary Figures and Tables

## Supplementary Material

Figure S1

## Supplementary Material

Figure S2
