# Supplementary material for: Acute stress is detrimental to heart regeneration in zebrafish
Source: Open Biol. 2016 Mar 30;6(3):160012. doi: 10.1098/rsob.160012 (PMC4821245; doi:10.1098/rsob.160012)
Supplement: Supplementary Figures and Tables [file rsob160012supp1.pdf]

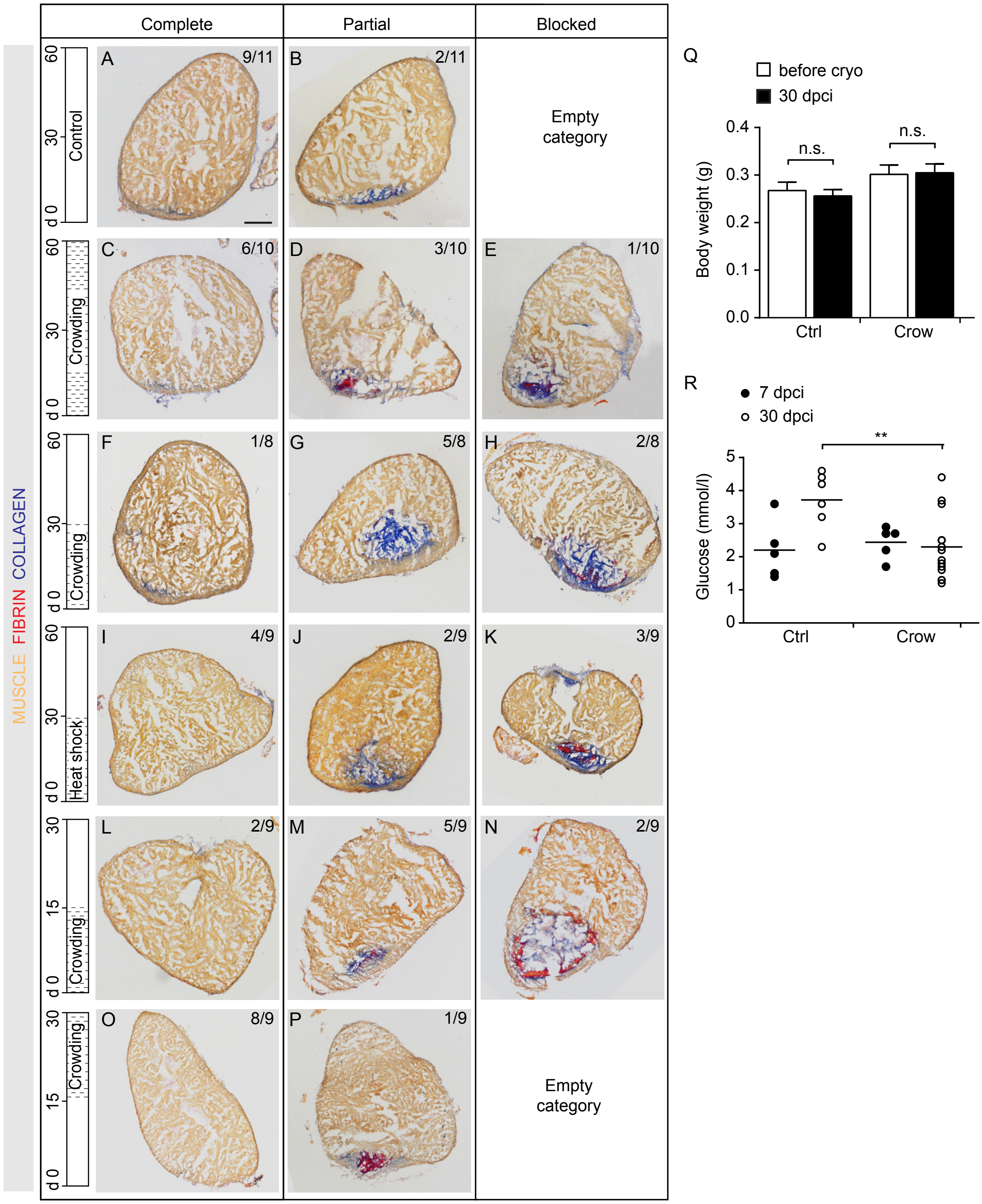

Figure S1.

The stress predominantly affects the first phase of heart regeneration.

(**A-K**) Heart sections at 60 dpci after AFOG staining were analyzed in the same manner as described in Fig. 2. After cryoinjury, zebrafish were subjected to daily stress either during the whole period of 60 days (crowding: C-E) or only during the first 30 days (crowding: F-H; heat shock: I-L). (**L-P**) Heart sections at 30 dpci after AFOG staining. In this case, zebrafish were exposed to daily crowding either during the first 2 weeks (L-N) or during the final 2 weeks (O, P). (**L-N**) The cryoinjured hearts of fish that were stressed for the first 2 weeks followed by 2 weeks of recovery at normal conditions display impaired regeneration. 78% of the animals displayed a partial or complete blockage of heart regeneration. (**O, P**) The cryoinjured hearts of fish that were kept at normal conditions for the first 2 weeks and then exposed to stress for the final 2 weeks, regenerated the hearts. The analysis of the regenerative process in the different groups revealed that daily exposure to stress has a stronger impact on the initial phase of heart regeneration. (**Q**) Analysis of the body weights before cryoinjuries and at 30 dpci revealed no significant weight changes during this period in control and stressed animals. (**R**) Glucose measurements revealed higher levels of blood glucose in the control animals as compared to stressed fish at 30 dpci. N≥8. No difference was observed at 7 dpci. Data are represented as mean ± SEM. **$P < 0.01$; N=5. Scale bar (A) = 100 μm.

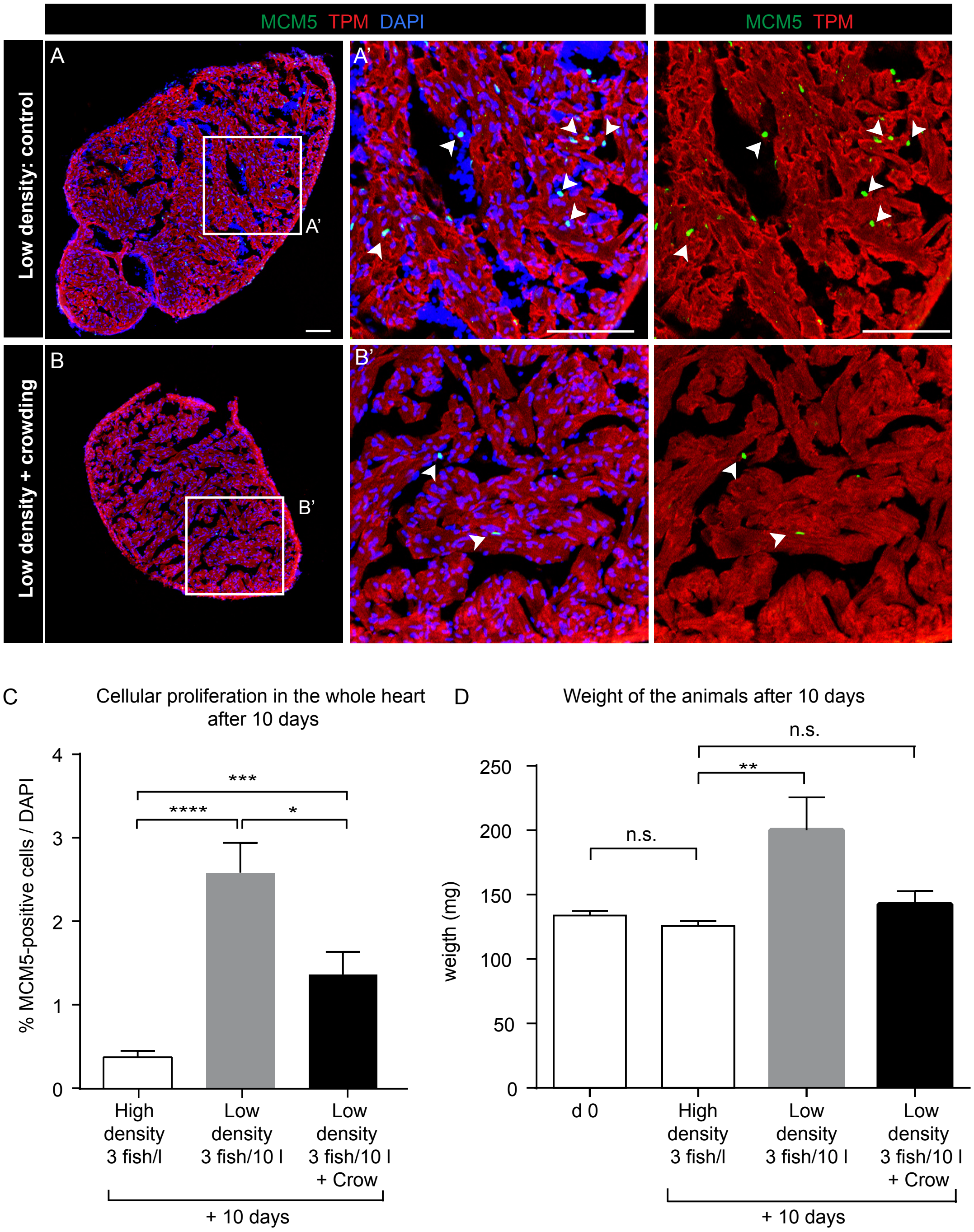

Figure S2.

Rapid cardiac growth is affected by daily acute stress exposure.

(**A, B**) Sections of hearts of control and stressed zebrafish 10 days after the transfer to low density conditions (3 fish / 10 liters) stained with antibodies against Tropomyosin (TPM, red), MCM5, a G1/S-phase marker (green) and with DAPI (blue). Juvenile zebrafish (2 months of age) were exposed 2 x per day to crowding (10 fish/ 250ml during 1h) during 10 days to investigate the effect of stress on cardiac homeostatic growth. (**A', B'**) Higher magnifications of the framed area shown in (A, B). A higher number of proliferating cardiac cells (CMs and non CMs) were identified in the control fish when compared to the stressed animals (arrows). (**C**) Bar graphs show the percentage of proliferating cells (MCM5-positive DAPI-positive / DAPI-positive in the tropomyosin-labeled myocardium) in the animals maintained at high density (5 fish per liter) and in control and stressed animals transferred to low density conditions (3 fish in 10 liters). Ventricles exposed to daily stress (10 fish/ 250 ml, 2 x 1h/day) showed a significantly lower proportion of proliferating cardiac cells than control hearts. (**D**) Bar graphs show the average weight of the animals at day 0, after 10 days in high density conditions (3 fish in 1 liter), after 10 days in low density conditions (3 fish in 10 liters) and after 10 days in low density conditions with daily exposure to crowding (10 fish/ 250 ml, 2 x 1h/day). Data are represented as mean ± SEM. *P < 0.05, **P < 0.01 ***P < 0.001, ****P < 0.0001; N≥4.

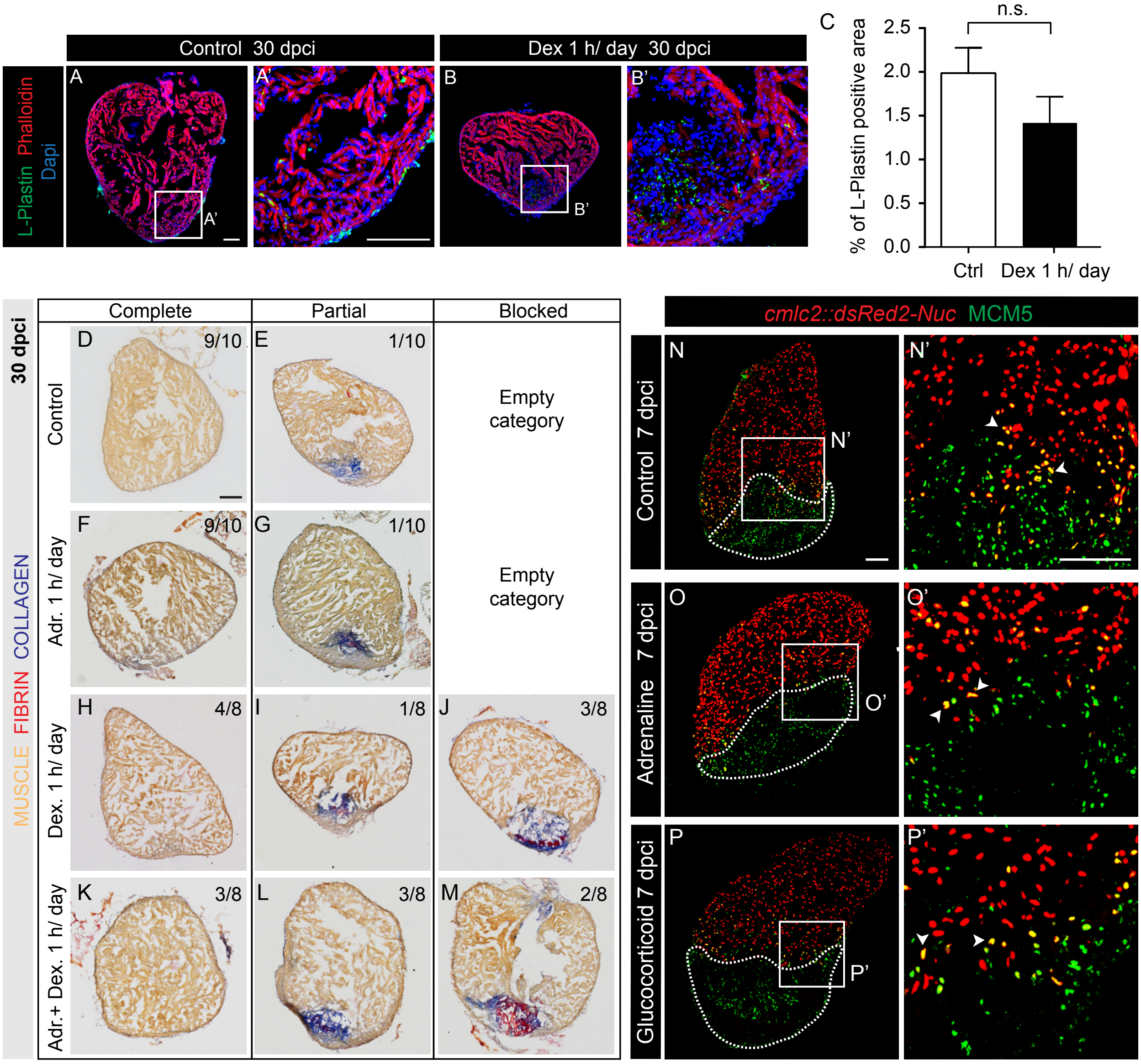

Figure S3.

The concomitant administration of dexamethasone and adrenaline after cryoinjury mimics the effect of stress on heart regeneration.

(**A, B**) Representative images of cryoinjured hearts at 30 dpci labeled with phalloidin (red, F-actin), dapi (blue) and antibody against L-Plastin (green, leukocytes). (**A', B'**) Higher magnification of the frame areas shown in (A, B). (**C**) Quantification of L-Plastin-positive area normalized to the total area of the ventricles revealed no significant effect of daily acute dexamethasone (2 mg/l) treatment on cardiac inflammation. N > 4. (**D-M**) Sections of hearts at 30 days post cryoinjury (dpci) after Aninlin blue acid Fuchsin Orange-G (AFOG). (**F-M**) The acute administration (1 hour per day) of dexamethasone (Dex, 2 mg/l) alone (H-J) or concomitantly with adrenaline (adr, 1 mg/l) (K-M) resulted in cardiac regenerative impairment in 50% and 62.5% of the fish, respectively. In contrast, animals treated with adrenaline alone during 1 hour per day displayed similar regenerative scores as the control (D-G). (**N-P**) Heart sections of *cmlc2::dsRed2-Nuc* transgenic zebrafish at 7 dpci immunostained against MCM5 (green). (**N'-P'**) Higher magnification of the framed areas shown in (N-P). Proliferating CMs could be identified in all groups by the overlap between MCM5 and DsRed (arrowheads). In contrast to adrenaline (1 mg/l, 1h /day), the treatment with glucocorticoid (hydrocortisone, 1 mg/l) lead to a reduction in CM proliferation at 7 dpci. Cryoinjured parts are encircled with a dashed line. Scale bar (A, D, N) = 100 µm.

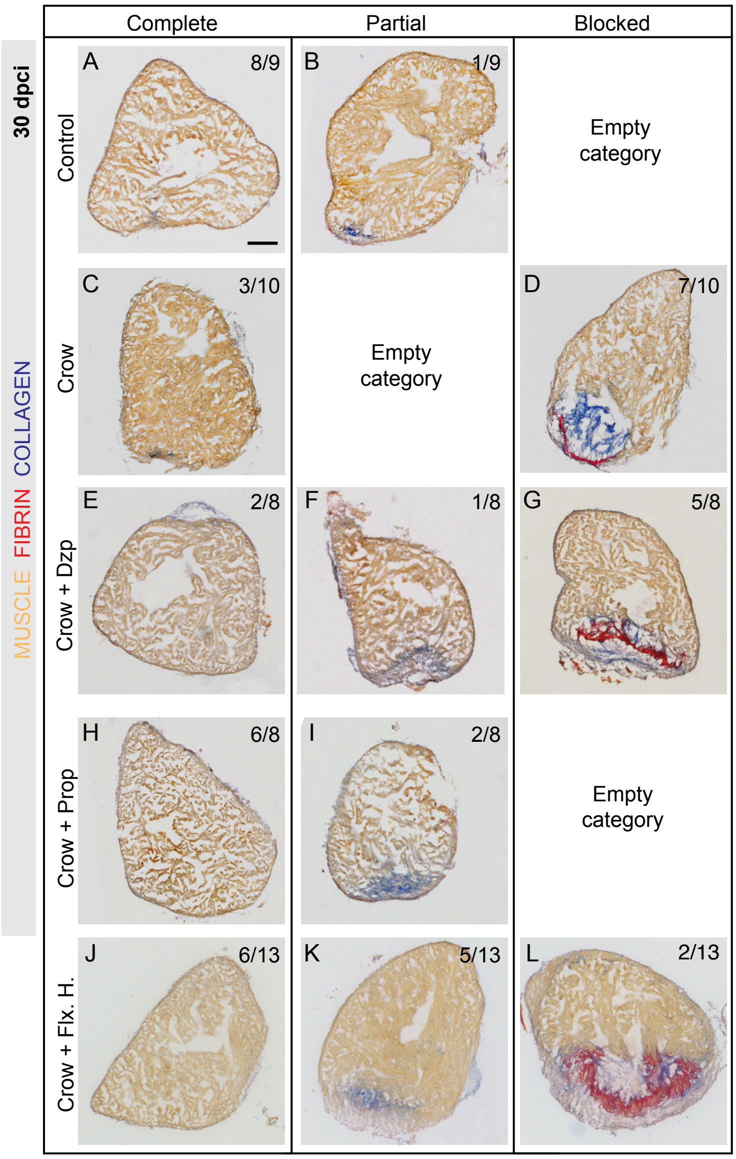

Figure S4.

Propranolol and fluoxetine hydrochloride administration have a beneficial effect on cardiac regeneration in the stressed animals.

(**A-L**) Heart sections at 30 dpci after AFOG staining. (**E-L**) The treatment of the stressed animals with propranolol (1 mg/l, 1 h/day, 3 days pretreatment, H, I) and fluoxetine hydrochloride (100 µg /l, continuous, 2 weeks pretreatment, J-L) had a significant rescue effect on the stress-induced regenerative impairment whereas the administration of diazepam (1 mg/l, continuous, 3 days pretreatment, E-G) did not rescue the negative effect of stress on heart regeneration. Scale bar (A) = 100 µm.

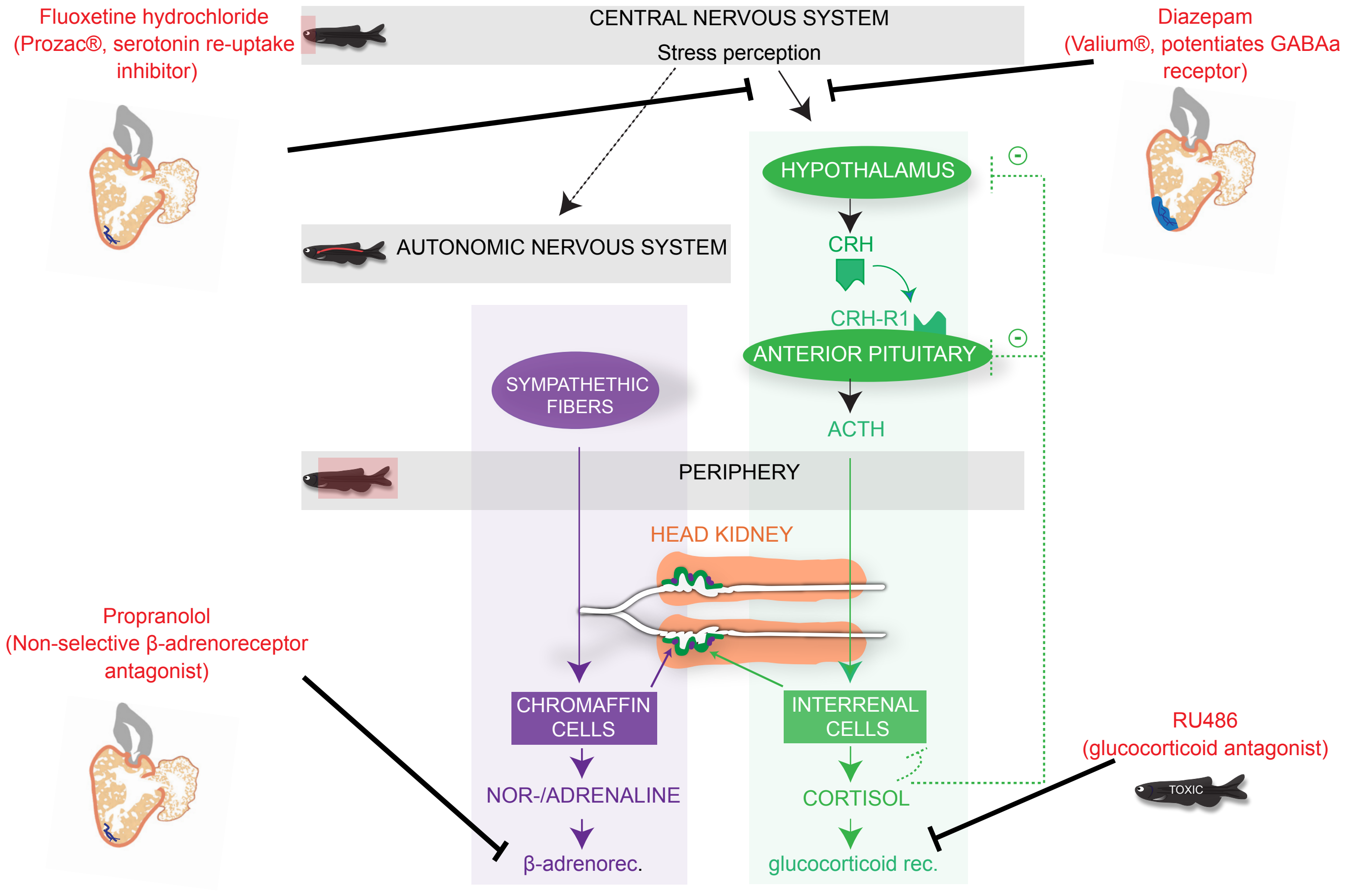

Figure S5.
Modulation of the stress response: effect on cardiac regeneration.
In zebrafish, exposure to different stressors activates two main axes: (1) the hypothalamus-pituitary interrenal (HPI) axis (green) and (2) the sympathetic-chromaffin cell axis (purple).
The treatment of the stressed animals after cryoinjury with fluoxetine hydrochloride (serotonin re-uptake inhibitor) and propranolol (non-selective β-adrenoreceptor antagonist) had a significant rescue effect on the stress-induced regenerative impairment. The administration of diazepam (GABAa receptor enhancer) did not show any beneficial effect on scar resorption. The intermittent adminsitration of RU486 (glucocorticoid antagonist) did not rescue cardiomyocyte proliferation in the stressed animals and the prolonged treatment with this drug was toxic and resulted in a high lethality.

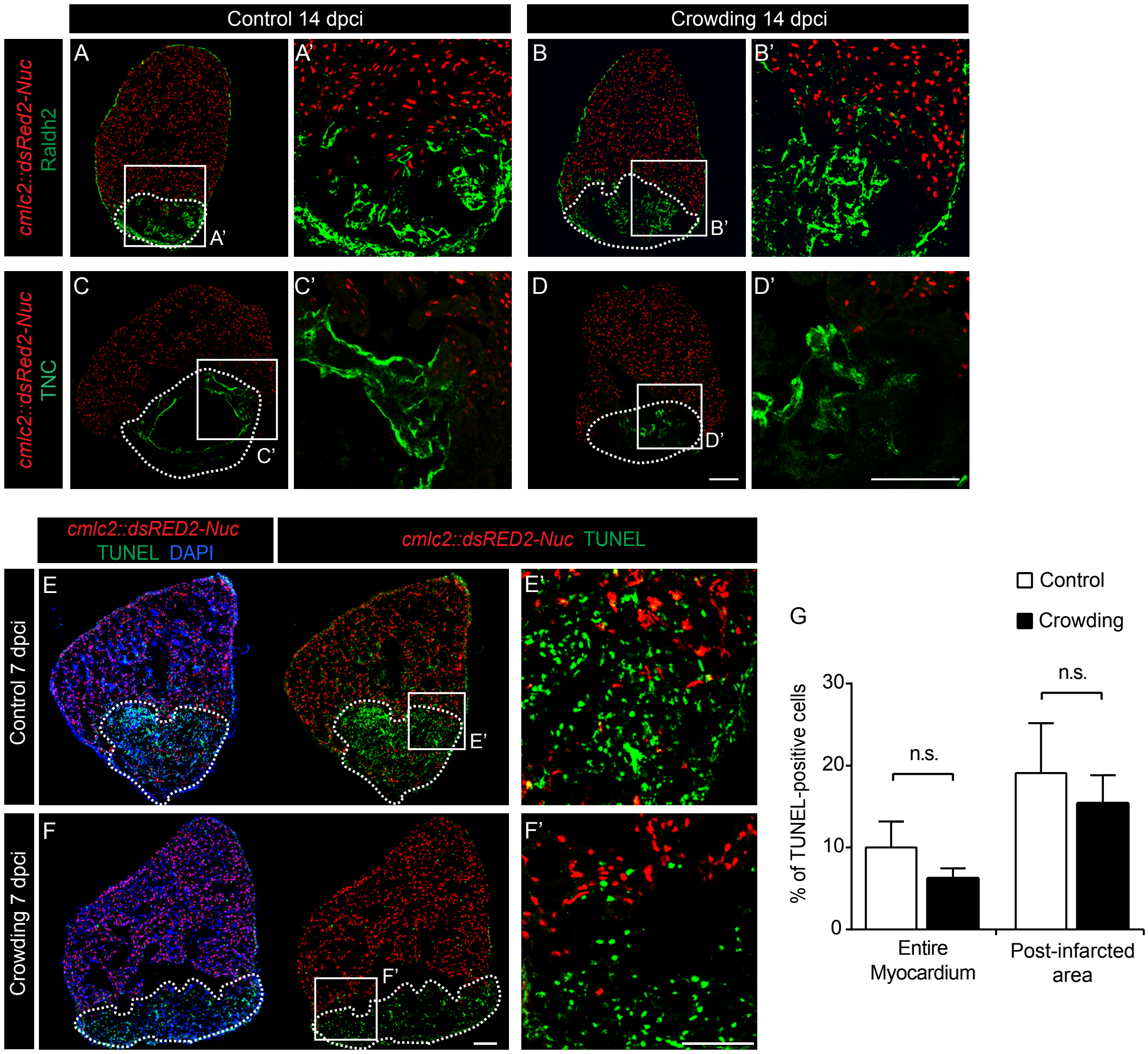

Figure S6.

Epi/endocardium activation, Tenascin C expression and cellular apoptosis are not affected by daily exposure to stress.

(**A-D**) Sections of *cmlc2::dsRed2-Nuc* hearts of control and stressed zebrafish at 14 dpci immunostained against the retinoic acid synthetizing enzyme (Raldh2, green), which is a marker of the activated epi-endocardium (A, B) or against Tenascin C (TNC, green), which is an ECM protein involved in tissue remodeling (C, D). (**A'-D'**) Higher magnifications of the framed areas shown in (A-D). Expression of the Raldh2 enzyme and Tenascin C could be detected at the injured site and in the epicardium both in control (A', C') and stressed (B', D') fish. This indicates no major impact of daily stress on epicardial/endocardial activation or on Tenascin C regulation. Scale bar (D, D') = 100 µm.

(**E, F**) Sections of *cmlc2::dsRed2-Nuc* hearts at 7 dpci after TUNEL assay and DAPI staining. (**E', F'**) Apoptotic cells were detected by signal overlap between TUNEL and DAPI in the cryoinjured parts but also in the rest of the ventricle. (**G**) Quantification of TUNEL and DAPI positive cells allowed to estimate the level of cellular apoptosis in the ventricle of the control and crowded zebrafish. No significant difference in apoptosis was observed between control and stressed fish at 7 dpci. Cryoinjured parts are encircled with a dashed line. Data are represented as mean ± SEM. Scale bar (F, F ') = 100 µm.

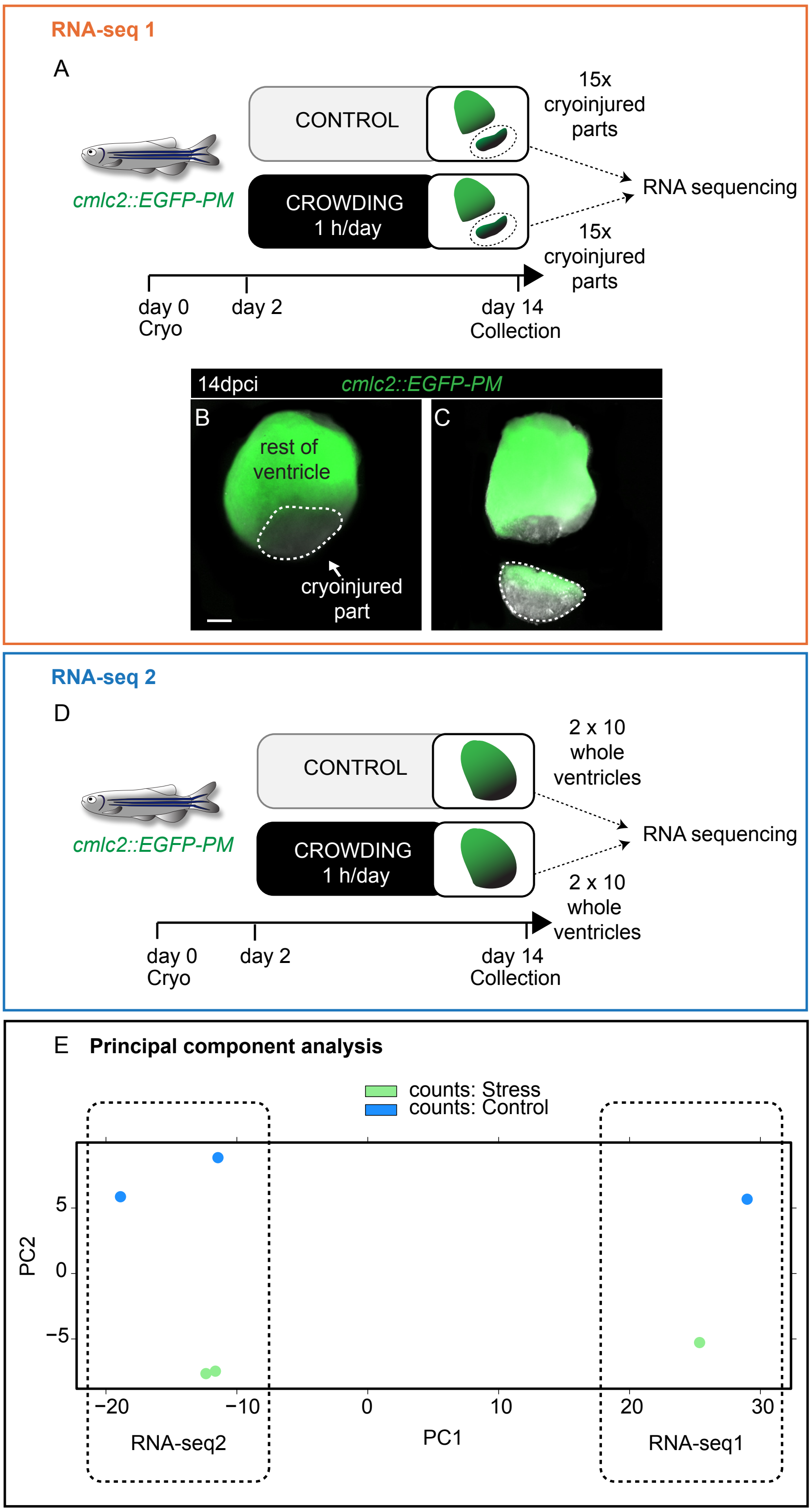

Figure S7.

Experimental setup for the RNA-seq1 & 2 experiments and principal components analysis of both experiments.

(**A**) Experimental setup for the RNA sequencing 1 performed at 14 dpci with the RNA extracted from the cryoinjured parts of the stressed and control hearts. (**B, C**) The use of *cmlc2::EGFP-PM* transgenic zebrafish, expressing GFP in the plasma membrane of CMs, enabled to identify and isolate the cryoinjured parts of the ventricle used for the RNA-sequencing experiment (15 cryoinjured parts were pulled together for each group in order to get sufficient amount of RNA). (**D**) Experimental setup for the RNA sequencing 2 performed at 14 dpci with the RNA extracted from the wholes ventricles of the stressed and control hearts. (**E**) Principal component analysis plot performed with RNA-seq1 and RNA-seq2. The Principal Compenent Analysis (PCA) is a classical method of statistical analysis. By transforming the set of observations into linearly uncorrelated variables called “principal component”. By definition the PCs are ordered by the variance they can explain in the data. PC1 explains the largest variance, then PC2, then PC3 etc. In our experiment, PC1 allows discriminating the two independant RNAseq experiments, and PC2 discriminates the stress and the control samples in both RNAseq experiments. Scale bar (B)= 100 µm.

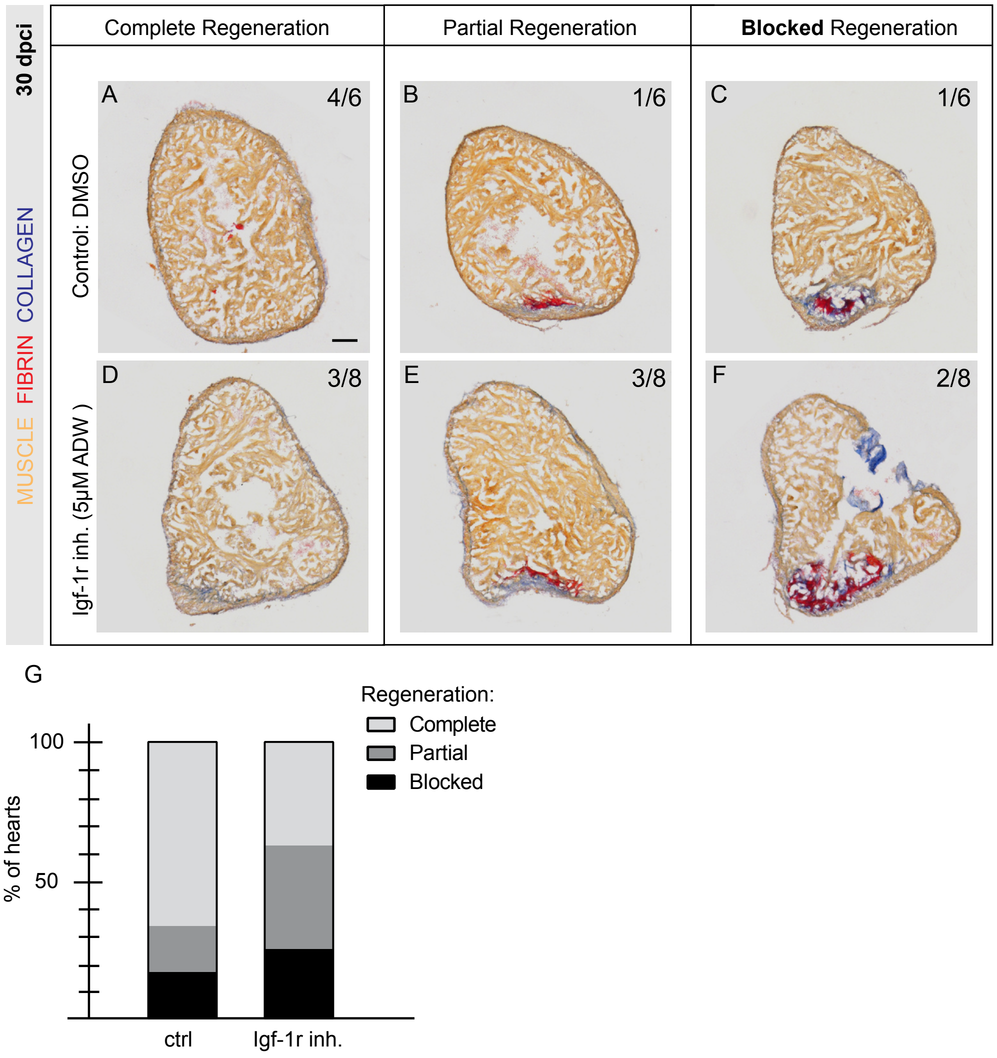

Figure S8.

Inhibition of IGF-1 receptor impairs heart regeneration.

(**A-F**) Heart sections at 30 dpci after AFOG staining. (**A-C**) In the control group treated with 0.05% DMSO, 66% of the zebrafish completely regenerated the heart at 30 dpci. (**D-F**) In the group treated with NVP-ADW742 (5 µM), a specific inhibitor of the Igf1r kinase activity, 62.5% of the fish showed impaired cardiac regeneration at 30 dpci. (**G**) Histograms represent the percentage of zebrafish hearts with complete (white), partial (gray) or blocked (black) regeneration at distinct experimental settings (regenerative scores). Scale bar (A) = 100 µm.

Name	# of Entities	Expanded # of Entities	# of Measured Entities	Median change	p-value	Hit type
Proteins Involved in Pathogenesis of Melanoma	245	262	180	1.121233899	5.5849E-06	Disease Collections
**Built Pathway_IGFBP1b_downstream cell processes**	178	763	238	1.086427761	2.7121E-05	Private pathways
Proteins Involved in Pathogenesis of Glioma	304	340	207	1.083054452	2.8003E-05	Disease Collections
Defective Clearance of Apoptotic Keratinocytes in Systemic Lupus Erythematosu	79	245	89	1.071944531	2.9092E-05	Disease Collections
**BMP7-ACVR2 Expression Targets**	27	29	21	1.610822919	0.00026135	Expression Targets Pathways
**TGFB1-ACVRL1 Expression Targets**	221	233	138	1.175402723	0.00028927	Expression Targets Pathways
EGF/CTNN Expression Targets	143	156	96	1.279815128	0.00032076	Expression Targets Pathways
**B Cell Activation**	62	841	506	1.181790244	0.00032227	Cell Signaling
TLR4/NF-kB/IRF Expression Targets	70	76	37	1.07553905	0.00034869	Expression Targets Pathways
Pathway_Genes_Imp	954	954	628	1.033202234	0.00037041	Private pathways
B-cell Chronic Lymphocytic Leukemia Overview	122	430	273	1.258365961	0.0003856	Disease Collections
**Extracellular Matrix Turnover**	36	166	76	1.228851937	0.00039486	Cell Process Pathways
Melanoma Overview	176	627	429	1.131958925	0.00043896	Disease Collections
EphrinR -> actin signaling	15	216	141	-1.020568772	0.00051062	Receptor Signaling
Dystrophin Glycoprotein Complex Signaling in Duchenne Muscular Dystrophy	67	792	490	1.151354255	0.00054743	Disease Collections
OXIDATIVE(ROS)Dystrophin Glycoprotein Complex Signaling in Duchenne Mus	67	792	490	1.151354255	0.00054743	Private pathways
Proteins Involved in Pathogenesis of Glioblastoma	188	210	138	-1.044766759	0.00055643	Disease Collections
Hodgkin Lymphoma Overview	146	614	285	1.254934272	0.00062251	Disease Collections
**TLR4 -> IRF signaling**	14	14	10	1.837768696	0.0007392	Receptor Signaling
**TGFB1-TGFBR1 Expression Targets**	89	91	47	1.269441599	0.00076762	Expression Targets Pathways
Proteins Involved in Pathogenesis of Cataract	95	95	53	1.106356331	0.00099064	Disease Collections
Role of Hexosamine Pathway in Diabetic Microangiopathy	26	145	71	1.1450458	0.0009942	Disease Collections
**T Cell Activation**	80	948	471	1.033202234	0.00101336	Cell Signaling
**PDGF/STAT Expression Targets**	80	89	57	1.164725658	0.00102698	Expression Targets Pathways
Proposed Mechanisms of Antiepileptic Effects of a Ketogenic Diet	56	365	219	1.285492956	0.00108017	Disease Collections
Atlas of Signaling	381	6035	3424	1.083054452	0.00113812	Cell Signaling
**Insulin/CEBPA/CTNNB/FOXA/FOXO Expression Targets**	145	171	109	-1.019580018	0.00129622	Private pathways
**Insulin/CEBPA/CTNNB/FOXA/FOXO Expression Targets**	145	171	109	-1.019580018	0.00129622	Expression Targets Pathways
**Notch_Insulin/CEBPA/CTNNB/FOXA/FOXO Expression Targets**	145	171	109	-1.019580018	0.00129622	Private pathways
**Cell Cycle Regulation**	135	2176	1436	1.13136165	0.00135125	Cell Signaling
Common Non-genomic Effects of Thyroid Hormones	60	338	195	-1.020568772	0.00140719	Disease Collections
BMP2 Activates WNT Signaling in Pulmonary Artery Smooth Muscle Cells	26	253	163	-1.028555619	0.00154104	Disease Collections
**TGFB1-TGFBR2 Expression Targets**	116	125	77	1.205982884	0.00157163	Expression Targets Pathways
**Actomyosin-Based Movement**	25	85	31	-1.305557659	0.00160695	Cell Process Pathways
**Actin Cytoskeleton Regulation**	51	546	369	1.030942621	0.00179848	Cell Signaling
Ca2+ Overload in Duchenne Muscular Dystrophy	59	256	150	1.033202234	0.00184601	Disease Collections
Role of HMGB1 and IL1B in Neuroinflammation in Epilepsy	25	29	19	-1.115450282	0.00186536	Disease Collections
**TNF/NF-kB Expression Targets**	127	135	68	1.164725658	0.00192162	Expression Targets Pathways
INHBA-ACVR2/ACVR1 Expression Targets	25	27	20	1.610822919	0.00193913	Expression Targets Pathways
**T-cell Receptor Signaling**	71	371	203	-1.00209964	0.00194978	Immunological Pathways
Onset of Atopic Dermatitis	72	343	203	-1.00209964	0.00194978	Disease Collections
**IL1B Expression Targets**	169	195	109	1.0215013	0.00196439	Expression Targets Pathways
Vascularization in Hepatocellular Carcinoma	25	101	52	1.077367242	0.00203218	Disease Collections
Secondary Glioblastoma	66	343	237	1.258365961	0.00204329	Disease Collections
ROS metabolism	45	108	16	1.888036854	0.00217801	Metabolic Pathways
EGF/MEF/MYOD/NFATC Expression Targets	145	231	138	1.03973094	0.00222991	Expression Targets Pathways
Leptin/STAT Expression Targets	96	107	63	1.194807312	0.00239132	Expression Targets Pathways
AGT/CREB Expression Targets	116	193	110	1.094417435	0.00244381	Expression Targets Pathways
Vitamin A (retinol) metabolism and visual cycle	93	165	19	1.22335471	0.00261586	Metabolic Pathways
Sister Chromatid Cohesion	27	174	113	1.317461315	0.00276692	Cell Process Pathways

Table S1.
Gene set enrichment analysis for the the fused RNA seq experiment (RNA-seq1&2) to highlight changes in gene expression between control and stressed zebrafish hearts. The table shows the 50 most significant **pathway** gene sets for the fused RNA-seq experiment: RNA-seq1 & RNA-seq2. The analysis was performed with Pathway Studio.

Name	# of Entities	Expanded # of Entities	# of Measured Entities	Median chang	p-value	Hit type
SRP-dependent cotranslational protein targeting to membrane	108	108	74	1.77234506	3.0145E-10	biological_process
viral transcription	82	82	56	1.84158913	4.4373E-10	biological_process
translational termination	89	89	62	1.7790759	1.112E-08	biological_process
viral life cycle	93	93	63	1.77234506	4.2407E-08	biological_process
translational elongation	149	149	74	1.76277199	3.1223E-07	biological_process
**cell adhesion**	658	658	342	1.03973094	1.4525E-06	biological_process
response to virus	145	145	68	1.37681504	1.67E-06	biological_process
cytosolic large ribosomal subunit	68	68	33	1.7790759	1.7438E-06	cellular_component
**actin binding**	395	395	223	-1.0781643	1.789E-06	molecular_function
Z disc	124	124	71	1.0368129	4.4501E-06	cellular_component
extracellular region	2319	2319	793	1.10562778	1.0497E-05	cellular_component
cytosol	2752	2752	1886	1.1016447	1.3491E-05	cellular_component
homophilic cell adhesion	160	160	47	-1.1749899	1.451E-05	biological_process
nuclear-transcribed mRNA catabolic process, nonsense-medi	122	122	86	1.64589637	1.7059E-05	biological_process
**extracellular matrix**	250	250	154	1.1450458	4.0576E-05	cellular_component
cytosolic small ribosomal subunit	59	59	28	1.78686236	5.2213E-05	cellular_component
proteinaceous extracellular matrix	318	318	167	1.12703243	8.9256E-05	cellular_component
DNA strand elongation involved in DNA replication	31	31	21	1.87578021	0.00011666	biological_process
**cytokine binding**	22	22	11	1.66175581	0.00011674	molecular_function
**structural constituent of muscle**	52	52	28	-1.22291476	0.00013793	molecular_function
innate immune response	669	669	341	1.09441743	0.0001406	biological_process
structural constituent of ribosome	416	416	112	1.5427905	0.00014143	molecular_function
cellular response to exogenous dsRNA	14	14	6	2.831489	0.00014868	biological_process
**myosin complex**	59	59	35	-1.37424361	0.00019363	cellular_component
heparin binding	166	166	75	1.10667467	0.0001945	molecular_function
**collagen binding**	61	61	42	1.36796593	0.00020382	molecular_function
inositol phosphate-mediated signaling	12	12	6	-2.02442083	0.00022145	biological_process
transmembrane signaling receptor activity	178	178	71	-1.02278562	0.00022579	molecular_function
muscle filament sliding	39	39	24	1.15324394	0.00022622	biological_process
positive regulation of neutrophil chemotaxis	19	19	11	2.51787823	0.00022765	biological_process
translational initiation	149	149	100	1.58878194	0.00024611	biological_process
actin filament binding	97	97	70	-1.08544637	0.00025982	molecular_function
response to vitamin D	27	27	19	-1.46988038	0.00031118	biological_process
protein homodimerization activity	765	765	434	1.03320223	0.00031824	molecular_function
RNA metabolic process	250	250	195	1.49149742	0.00042772	biological_process
cellular response to mechanical stimulus	86	86	52	-1.04631187	0.00050118	biological_process
mRNA metabolic process	227	227	180	1.52096928	0.0005202	biological_process
**mitotic cell cycle**	394	394	300	1.27847668	0.00060125	biological_process
**cardiac muscle cell action potential involved in contractic**	10	10	7	-1.96299321	0.00063621	biological_process
positive regulation of potassium ion transport	16	16	9	-1.66351809	0.00075449	biological_process
**myoblast migration**	5	5	5	2.45266796	0.00081209	biological_process
superoxide anion generation	15	15	8	3.21042632	0.00085169	biological_process
immune response	449	450	119	1.15294089	0.00099846	biological_process
**regulation of stress fiber assembly**	7	7	6	-1.99257912	0.00100537	biological_process
cellular amino acid metabolic process	50	50	32	1.12440149	0.00108476	biological_process
viral process	320	320	243	1.42239843	0.00110895	biological_process
cell periphery	48	48	28	1.09441743	0.00116032	cellular_component
regulation of small GTPase mediated signal transduction	174	174	108	-1.11122585	0.0012402	biological_process
small ribosomal subunit	48	48	18	1.72612426	0.00128253	cellular_component
energy reserve metabolic process	103	103	68	-1.11351284	0.00129081	biological_process

Table S2. Gene set enrichment analysis for the fused RNA seq experiment (RNA-seq1&2) to highlight changes in gene expression between control and stressed zebrafish hearts. The table shows the 50 most significant **GO-term** gene sets for the fused RNA-seq experiment: RNA-seq1 & RNA-seq2. The analysis was performed with Pathway Studio.

geneID	log2FC_fused	padj_fused	type of RNA
IFITM1	3.742286001	1.14E-30	
ENSDARG00000084533	3.881034115	1.21E-28	miRNA
ENSDARG00000081938	8.047716049	1.79E-24	Mt-tRNA
SLC25A4	-2.760180063	1.18E-21	
ENSDARG00000088865	3.443302383	1.18E-21	miRNA
LGALS3BP	3.113514297	1.69E-21	
CORO2A	-5.652379587	3.78E-21	
ENSDARG00000088976	3.582835203	3.15E-20	miRNA
ENSDARG00000086686	3.47104141	4.73E-20	miRNA
FHL2	2.929914201	1.73E-19	
ENSDARG00000089384	4.183491967	1.96E-19	miRNA
ENSDARG00000070212	3.256322883	1.13E-18	
ENSDARG00000090280	3.435020079	4.47E-18	miRNA
ENSDARG00000091738	3.373336585	6.97E-18	miRNA
PARP6	-2.569819142	1.76E-17	
MYBPH	-2.572453077	8.25E-17	
ENSDARG00000093902	7.612314957	3.87E-16	pseudogene
PCOLCE	2.707511492	1.34E-15	
GRIN3A	-4.873753905	1.85E-15	
ENSDARG00000087783	3.665121865	1.85E-15	miRNA
ENSDARG00000088564	3.429343718	1.85E-15	miRNA
IFI27	2.641037412	3.49E-14	
ADCY6	-2.924964698	3.70E-14	
CPAMD8	3.645294968	1.08E-13	
ENSDARG00000089068	7.09582739	2.01E-13	pseudogene
MIH7B	-2.872828154	3.78E-13	
ENSDARG00000090619	2.873602128	3.78E-13	miRNA
ENSDARG00000086606	7.03980247	3.78E-13	pseudogene
ENSDARG00000086256	-2.733709962	4.41E-13	protein_coding
CRIP1	2.06748148	4.84E-13	
GNB3	-5.560717107	5.66E-13	
ENSDARG00000086396	2.928302686	8.23E-13	miRNA
SEC61G	2.213266624	8.24E-13	
ENSDARG00000086085	3.124629975	9.25E-13	miRNA
IFI27L1	2.450376839	1.06E-12	
ENSDARG00000088436	2.770051212	1.22E-12	protein_coding
ENSDARG00000078859	4.727957055	1.46E-12	protein_coding
MYRFL	-2.53518149	1.89E-12	
ENSDARG00000087953	3.056798055	1.97E-12	miRNA
ENSDARG00000089382	2.864534502	3.65E-12	protein_coding
PDZRN3	-2.361487556	5.04E-12	
SLC16A1	-2.31344781	6.27E-12	
BMP10	-2.566374785	7.56E-12	
ENSDARG00000096403	2.7441639	7.94E-12	lincRNA
ENSDARG00000070873	2.558192575	1.05E-11	protein_coding
ENSDARG00000090733	2.774640053	1.83E-11	miRNA
ENSDARG00000087337	2.801632112	1.98E-11	miRNA
HEPACAM2	6.641683192	1.98E-11	
ENSDARG00000017246	-2.401867587	3.29E-11	protein_coding
ENSDARG00000090146	2.984329301	4.74E-11	miRNA
RBP4	2.002717065	6.27E-11	
ENSDARG00000090175	2.882947206	6.63E-11	miRNA
ENSDARG00000082789	2.100222947	8.53E-11	Mt_tRNA
ENSDARG00000086192	3.098469469	1.09E-10	miRNA
KCNMB2	-2.581176089	1.79E-10	
ENSDARG00000015815	-1.809270076	2.43E-10	protein_coding
NR4A1	-2.146381259	3.12E-10	
EPHB3	-2.249748285	3.19E-10	
SULT2B1	2.547289475	3.56E-10	
ENSDARG00000058348	2.976849528	3.92E-10	protein_coding
ENSDARG00000088311	2.795463059	4.35E-10	miRNA
ETV4	2.433975283	5.45E-10	
ENSDARG00000088838	2.703044112	5.80E-10	miRNA
ENSDARG00000085168	6.285124238	6.35E-10	miRNA
ENSDARG00000097100	-2.044045148	6.70E-10	protein_coding
ENSDARG00000087747	6.227650516	1.08E-09	pseudogene
ENSDARG00000088582	6.225175014	1.09E-09	pseudogene
LGR4	-2.076095077	1.14E-09	

Table S3. Table showing the 50 most significant DE **non-coding RNAs**(miRNAs are highlighted in red) for the fused RNA-seq experiment: RNA-seq1 & RNA-seq2. The analysis was performed with Pathway Studio.
