# Supplementary material for: Acute stress is detrimental to heart regeneration in zebrafish
Source: Open Biol. 2016 Mar 30;6(3):160012. doi: 10.1098/rsob.160012 (PMC4821245; doi:10.1098/rsob.160012)
Supplement: Figure S1 [file rsob160012supp2.pdf]

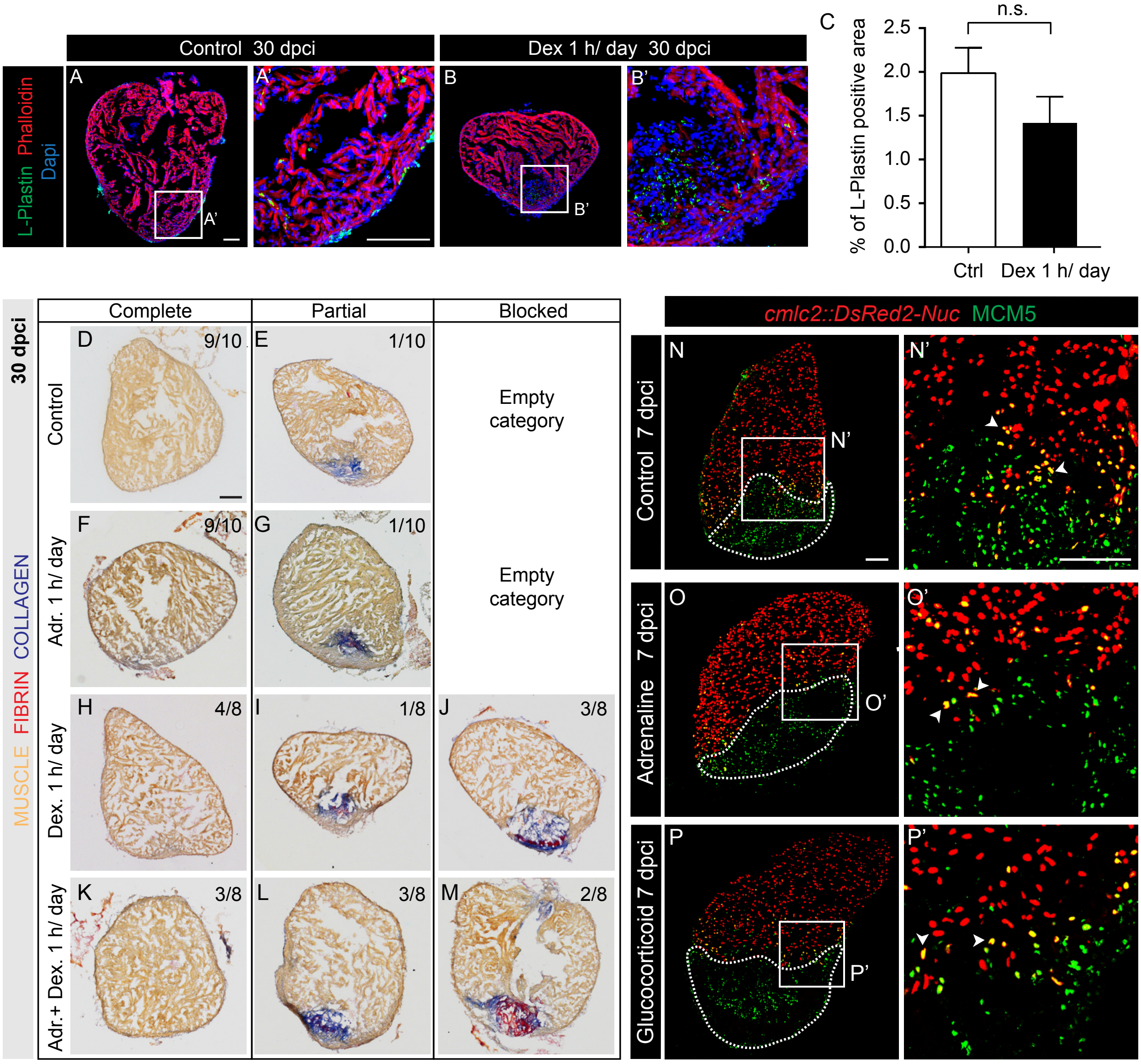

Figure S3.

The concomitant administration of dexamethasone and adrenaline after cryoinjury mimics the effect of stress on heart regeneration.

(**A, B**) Representative images of cryoinjured hearts at 30 dpci labeled with phalloidin (red, F-actin), dapi (blue) and antibody against L-Plastin (green, leukocytes). (**A', B'**) Higher magnification of the frame areas shown in (A, B). (**C**) Quantification of L-Plastin-positive area normalized to the total area of the ventricles revealed no significant effect of daily acute dexamethasone (2 mg/l) treatment on cardiac inflammation. N > 4. (**D-M**) Sections of hearts at 30 days post cryoinjury (dpci) after AFOG staining. (**F-M**) The acute administration (1 hour per day) of dexamethasone (Dex, 2 mg/l) alone (H-J) or concomitantly with adrenaline (adr, 1 mg/l) (K-M) resulted in cardiac regenerative impairment in 50% and 62.5% of the fish, respectively. In contrast, animals treated with adrenaline alone during 1 hour per day displayed similar regenerative scores as the control (D-G). (**N-P**) Heart sections of *cmlc2::DsRed2-Nuc* transgenic zebrafish at 7 dpci immunostained against MCM5 (green). (**N'-P'**) Higher magnifications of the framed areas shown in (N-P). Proliferating CMs could be identified in all groups by the overlap between MCM5 and DsRed (arrowheads). In contrast to adrenaline (1 mg/l, 1h /day), the treatment with glucocorticoid (hydrocortisone, 1 mg/l) lead to a reduction in CM proliferation at 7 dpci. Cryoinjured parts are encircled with a dashed line. Scale bar (A, D, N) = 100 µm.
