# Supplementary material for: Acute stress is detrimental to heart regeneration in zebrafish
Source: Open Biol. 2016 Mar 30;6(3):160012. doi: 10.1098/rsob.160012 (PMC4821245; doi:10.1098/rsob.160012)
Supplement: Figure S2 [file rsob160012supp3.pdf]

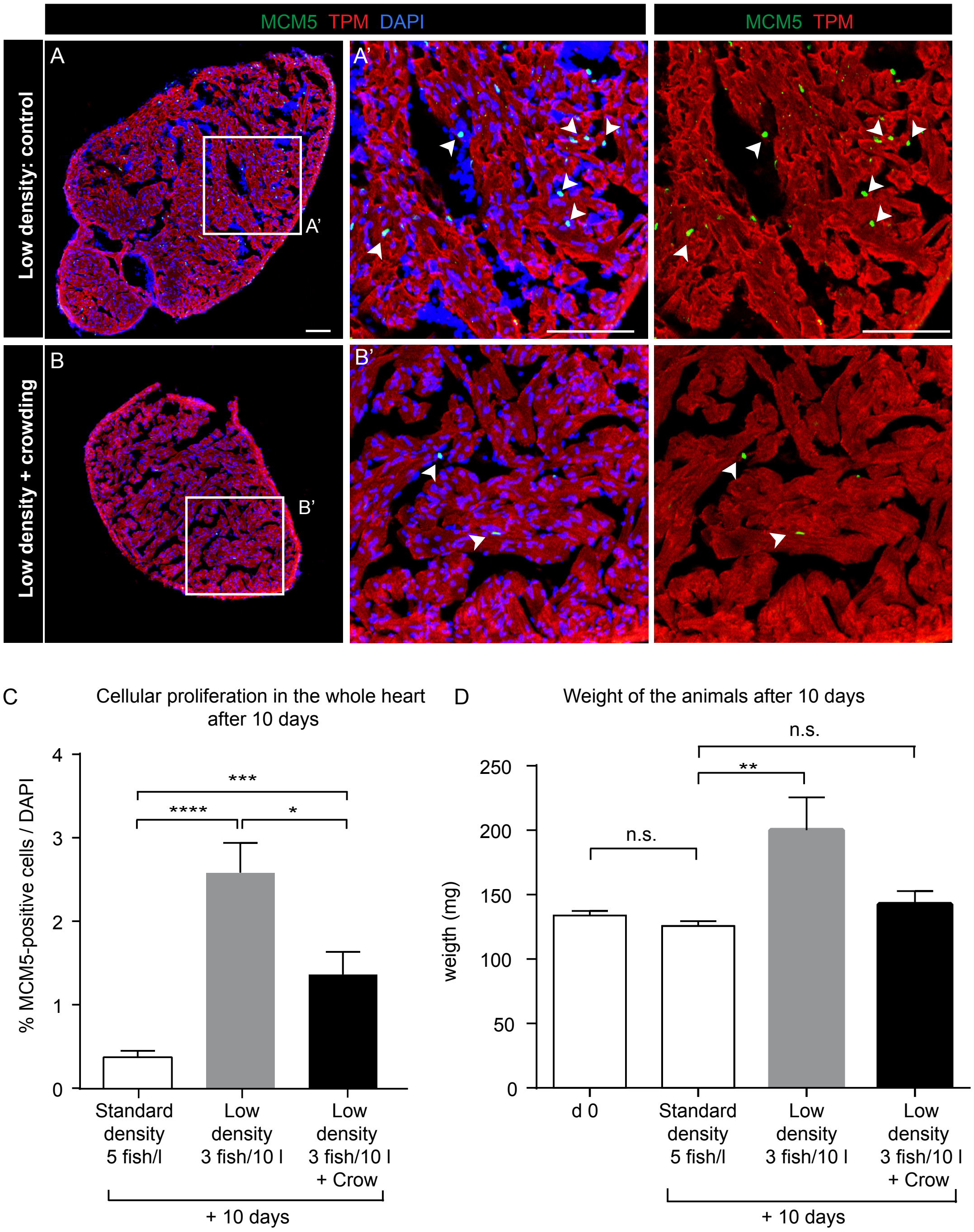

Figure S2.

Rapid cardiac growth is affected by daily acute stress exposure.

(**A, B**) Sections of hearts of control and stressed zebrafish 10 days after the transfer to low density conditions (3 fish / 10 liters) stained with antibodies against Tropomyosin (TPM, red), MCM5, a G1/S-phase marker (green) and with DAPI (blue). Juvenile zebrafish (2 months of age) were exposed 2 x per day to crowding (10 fish/ 250ml during 1h) during 10 days to investigate the effect of stress on cardiac homeostatic growth. (**A', B'**) Higher magnifications of the framed area shown in (A, B). A higher number of proliferating cardiac cells (CMs and non CMs) were identified in the control fish when compared to the stressed animals (arrows). (**C**) Bar graphs show the percentage of proliferating cells (MCM5-positive DAPI-positive / DAPI-positive in the tropomyosin-labeled myocardium) in the animals maintained at standard density (5 fish per liter) and in control and stressed animals transferred to low density conditions (3 fish in 10 liters). Ventricles exposed to daily stress (10 fish/ 250 ml, 2 x 1h/day) showed a significantly lower proportion of proliferating cardiac cells than control hearts. (**D**) Bar graphs show the average weight of the animals at day 0, after 10 days in standard density conditions (5 fish in 1 liter), after 10 days in low density conditions (3 fish in 10 liters) and after 10 days in low density conditions with daily exposure to crowding (10 fish/ 250 ml, 2 x 1h/day). Data are represented as mean ± SEM. $^*P < 0.05$, $^{**}P < 0.01$ $^{***}P < 0.001$, $^{****}P < 0.0001$; N≥4.
